# Characterization of Growth Suppressive Functions of a Splice Variant of Cyclin D2

**DOI:** 10.1371/journal.pone.0053503

**Published:** 2013-01-10

**Authors:** Karim Wafa, Jessica MacLean, Feixiong Zhang, Kishore B. S. Pasumarthi

**Affiliations:** Department of Pharmacology, Dalhousie University, Halifax, Nova Scotia, Canada; Instituto Gulbenkian de Ciência, Portugal

## Abstract

We have recently cloned a novel splice variant of cyclin D2 termed as cycD2SV. CycD2SV overexpression in several immortalized cell lines led to formation of ubiquitinated protein aggregates accompanied by a significant decrease in cell proliferation. Based on immuno co-localization and ultrastructural analysis experiments, cycD2SV protein aggregates were frequently found in various subcellular compartments such as endosomes, autophagosomes, lysosomes and the microtubule organizing centre. Secondary structure analysis revealed that the amino terminal α-helix in cycD2SV is not tightly packed with the cyclin box suggesting a misfolded conformation compared to other cyclins. Deletion analysis suggests that 1–53 amino acid region of cycD2SV may be required for protein aggregation and 54–136 amino acid region may mediate cell cycle inhibition. Based on co-immunoprecipitation experiments, we have shown that cycD2SV binds to cycD2 as well as CDK4. In addition, gene expression analysis demonstrated an upregulation in GADD45α and dynamin 2 mRNA levels in cycD2SV overexpressing cells. These two proteins are known to play critical roles in the DNA damage response and apoptosis pathways. TUNEL experiments were negative for apoptosis, however, cycD2SV expressing cells were more sensitive to cell death induced by external stressors such as trypsinization. Collectively our results suggest that cycD2SV mediates cell cycle inhibition by sequestering endogenous cell cycle proteins, such as cycD2 and CDK4, and possibly targeting them for ubiquitin mediated protein degradation.

## Introduction

Cell cycle progression in mammalian cells is dependent on interactions between cyclins and cyclin dependent kinases (CDKs) [1]. Specifically, mitogenic signals stimulate the expression of D-type cyclins (cycD1, D2 and D3) which bind to CDK4 and CDK6 [1]. Upon binding, the complex translocates to the nucleus where it phosphorylates the retinoblastoma protein (pRb). In an unphosphorylated state, the pRb protein binds and inactivates the transcription factor E2F. Once phosphorylated, pRb dissociates from E2F, allowing it to upregulate genes necessary for S-phase entry such as cycE, cycA, and CDK1 among other genes [1].

CycD2SV is a newly discovered truncated splice variant of cycD2 which shares the first 136 amino acids of cycD2 with a unique 20 amino acid carboxy terminal (CT) sequence [2]. Given the sequence similarity between cycD2SV and cycD2, it is possible that cycD2SV is also a positive regulator of the cell cycle. In support of this hypothesis, a recent study by Denicourt *et al.* demonstrated that cycD2SV, in conjunction with H-Ras, acts as a potent transforming protein compared to cycD2 [3]. However, they have not directly tested the effects of cycD2SV alone on cell cycle regulation. We have recently reported that ectopically expressed cycD2SV can form protein aggregates in embryonic cardiomyocytes and induce cell cycle arrest [4]. It is possible that cell type differences may account for the discrepancy between these two studies.

In this study, we investigated the function of cycD2SV in multiple immortalized cell lines. Consistent with the results obtained in primary cardiomyocytes, cycD2SV formed several small protein aggregates throughout cytoplasmic and nuclear compartments and significantly reduced cell cycle activity in T47D, NIH-3T3, HEK293 and MCF7 cells. Further, cycD2SV aggregation and cell cycle arrest phenotypes were only partially rescued by coexpression of CDK4. In stark contrast, cycD2 overexpression frequently led to formation of a single large perinuclear puncta followed by prompt cell cycle exit and these phenotypes were completely rescued by CDK4 coexpression. We demonstrated that cycD2SV is able to bind to CDK4 and cycD2, interfere with their function and possibly target them for ubiquitin mediated degradation. Further, cycD2SV expression is associated with impaired endoplasmic reticulum associated protein degradation (ERAD) and increased autophagic responses. In addition, we report that the cycD2SV cell cycle inhibition domain is present in the 54–136 amino acid sequence of the protein.

## Materials and Methods

### Cell Culture and Transient Transfections

All cell lines were purchased from the American Type Culture Collection (ATCC, Virginia). Cells were cultured in Dulbecco modified Eagle’s medium (DMEM, Wisent, Saint-Bruno, Quebec) supplemented with 10% fetal bovine serum (10% FBS-DMEM). Cells were seeded at 600,000 cells in 100 mm dishes, and at 150,000 in 35 mm dishes at day 0. At day 2, cells were transfected with expression constructs using Lipofectamine™ 2000 according to manufacturer’s instructions (Invitrogen, Burlington, Ontario). Cells were incubated with the transfection mixture for 5 hours and subsequently maintained in freshly added 10% FBS-DMEM for 18 hours post transfection, unless otherwise stated. Transfection efficiency was routinely determined by EGFP-C1 transfections where, on average, it was found to be between 50–60%. Cells plated in 100 mm dishes received 4 µg of DNA and 10 µl of Lipofectamine™ 2000 reagent, while cells seeded in 35 mm dishes received 1.7 µg of DNA and 4.3 µl of Lipofectamine™ 2000 reagent. For co-transfection of two expression constructs, cells were transfected at a 1∶1 ratio of the DNA constructs, such that 100 mm dishes received 2 µg, and 35 mm dishes received 0.425 µg of each construct. All cell culture reagents were purchased from Invitrogen (Burlington, Ontario).

### Cloning and Generation of Expression Constructs

Generation of mouse cycD2SV and cycD2 expression constructs with or without C-terminal fusions to a myc epitope (pcDNA-cycD2SVmyc, pcDNA-cycD2SV and pcDNA-cycD2myc) was previously described [4]. Generation of the CDK4 expression construct was previously described [4]. The EGFP-D2SV was generated by amplifying cycD2SV fragment using D2SVS4 and D2altAS primers (Table S1) from cycD2SV cDNA and cloning the fragment in CMV-EGFP-C1 vector (Clontech, Mountain View, California) in frame with EGFP. CycD2SVΔCTmyc, cycD2SV 1–53 and cycD2SV 54–136 myc were generated by amplifying 1–136, 1–53 and 54–136 portion of cycD2SV from TA-cycD2SV construct using appropriate primer pairs (Table S1). The fidelities of all constructs were confirmed by Southern blotting and or DNA sequencing (Robarts Research Institute, London, Ontario). The following constructs were received as generous gifts: TCRα-GFP (Dr. John Christianson, Stanford University, [5]), cycB1 (Dr. Karl Riabwol, University of Calgary, [6]), mcherry-p62 (Dr. **Terje Johansen**, University of Tromsø, [7], [8]), YFP-intersectin (Dr. John P. O’Bryan, University of Illinois, [9]) and HA-ubiquitin (Dr. James Fawcett, Dalhousie University).

### Immunofluorescence Staining

Cells were plated on coverslips (22×22 mm 0.08–0.13 mm thickness, VWR, Mississauga, Ontario) in 35 mm dishes, and transfected as described earlier. Cells were fixed in methanol for 15 minutes, permeabilized in 0.1% Triton X-100 for 5 minutes, and blocked with blocking buffer (1% v/v bovine serum albumin (BSA), 10% v/v goat serum in PBS) for one hour. Cells were probed with primary antibodies raised against myc (sc-40), cycD2SV, HA (sc-805), CDK4 (sc-260), cycB1 (sc-245), cycD2 (sc-593), and γ-tubulin (sc-10732) for one hour at 25°C, followed by a one hour incubation with secondary goat anti-mouse antibodies, conjugated to Alexa Fluor 488 or goat anti-rabbit antibodies conjugated to Alexa Fluor 555 dye (Invitrogen, Burlington, Ontario) for one hour. Subsequently, cells were incubated with 10 mg/ml Hoechst 33342 nuclear stain (Invitrogen, Ontario) for five minutes, washed extensively in cold PBS and mounted on glass slides using 1% w/v propyl 3,4,5-trihydroxybenzoate (propyl gallate) in a 1∶1 PBS/glycerol solution. Primary antibodies were diluted 1∶50, and secondary antibodies were diluted 1∶200 in block buffer unless otherwise stated. Generation of cycD2SV homemade antibodies was previously described [4]. All other antibodies were purchased from Santa Cruz Biotechnology Inc. Images were captured using a Leica DM2500 fluorescence microscope, fitted with a DFC500 digital acquisition system (Leica Microsystems, Concord, Ontario).

### [^3^H]-thymidine Labeling and Autoradiography

Transfected cells were maintained for twelve hours and pulsed with [^3^H]-thymidine (GE Healthcare Life Sciences, New Jersey) at a concentration of 1.0 µCi per 1 ml of medium for six or twenty-four hours at 37°C. Cells were fixed in cold methanol for fifteen minutes and processed for immunofluorescence as described earlier. Coverslips were air dried, coated with Kodak autoradiography emulsion type NTB (MarketLINK Scientific, Burlington, Ontario) and placed in a light-tight box at 4°C for 3 days. Coverslips were developed in Kodak-D19 developer (Sigma-Aldrich, Oakville, Ontario) for four minutes, washed in double distilled water, fixed with Ilford rapid fixer (Polysciences, Pennsylvania) for four minutes, and mounted on glass slides using propyl gallate solution. Cellular morphology was examined under bright field, and nuclei were identified with epi-fluorescence microscopy. Cells containing more than fifteen nuclear silver grains were identified as cells undergoing DNA synthesis.

### Protein Extraction, Immunoblotting and Immunoprecipitation

Transfected cells were harvested in tumor lysis buffer (1% NP40/Igpal, 5 mM EDTA, 50 mM Tris HCl pH 8.0, 10 mM phenylmethylsulphonyl fluoride (PMSF) and 1 mM Aprotinin), sonicated and centrifuged at 13,300 rpm for fifteen minutes at 4°C. The cytosolic fraction was collected, and protein concentration was determined using Bradford assay (Thermo Fisher Scientific, Nepean, Ontario) as indicated by the manufacturer. Equal amounts of protein (40–60 µg) were denatured in Lamelli buffer and resolved in 12.5% polyacrylamide gels at 100 volts. The resolved samples were electrophoretically transferred from the gel to Hybond ECL nitrocellulose membranes (GE Healthcare Life Sciences, New Jersey) by applying a constant current at 100 volts for one hour. The membrane was rinsed with distilled water for five minutes and blocked for one hour in PBS containing 0.1% Tween 20, 5% skimmed milk powder and 3% BSA. The blots were incubated for one hour with primary antibodies specific for the following proteins: cycD2SV (1∶500), cycD1 (sc-753, 1∶500), cycD2 (sc-593, 1∶500), p27 (sc-528, 1∶500), CDK4 (sc-260, 1∶10,000), α-tubulin (sc-8035, 1∶5000), and c-myc (sc-40). The blots were washed in 0.1% Tween 20-PBS and incubated with goat-anti rabbit, or goat-anti mouse secondary antibodies conjugated to horse radish peroxidase (HRP) for one hour. Protein bands were detected by ECL Plus Western Blotting Detection System via the chemiluminescence method according to manufacturer’s instructions (GE Healthcare Life Sciences, New Jersey).

For immunoprecipitation, cells were lysed in tumor lysis buffer eighteen hours post transfection and 500 µg of protein was incubated with 0.5–1 µg of cycD2SV, cycD2 or myc antibodies for seventeen hours at 4°C, followed by the addition of protein-A-Sepharose beads (GE Healthcare Life Sciences, New Jersey) for one hour at 4°C. Immunocomplexes bound to the beads were collected by centrifugation at 3,000 rpm for one minute, resuspended in Lamelli buffer and boiled at 95°C for five minutes. The immunoprecipitated samples were resolved on 12.5% SDS-PAGE gel, electrophoretically transferred to Hybond ECL nitrocellulose membranes and processed for chemiluminescence detection as described earlier.

### Apoptosis Assay

Transfected cells seeded on coverslips were fixed with freshly prepared 4% paraformaldehyde in PBS for one hour at room temperature, washed with PBS and permeabilized in 0.1% triton X-100 and 0.1% sodium citrate for two minutes at 4°C. Cells were processed for TUNEL staining according to the manufacturer’s instructions (Roche in Situ Cell Death Detection Kit, TMR red). TUNEL positive cells were counted using fluorescence microscopy.

### Fluorescence Activated Cell Sorting and QPCR Array Analysis

HEK293 cells seeded in 100 mm dishes were transfected with EGFP-D2SV or EGFP-C1 control plasmid for six hours, and grown under subconfluent conditions. After 48 hours, cells were trypsinized, centrifuged at 2,000 rpm for two minutes, and resuspended in PBS. To eliminate cell clumps, cells were passed through a 40 µm mesh filter. To prepare for fluorescence activated cell sorting (FACS), cells were centrifuged again and resuspended in 1 ml of sorting buffer (PBS containing 15 mM Hepes, 1 mM EDTA, 0.5% BSA). Transfected cultures were sorted for EGFP-C1 or EGFP-D2SV cell populations using a BD FACSCanto II flow cytometer. Sorted cells were pelleted by centrifugation at 2,000 rpm for two minutes and cell pellets were processed for RNA extraction using an RNeasy PLUS kit (Qiagen, Mississauga, Ontario). In brief, lysed cells underwent a series of filtration and washing steps in filter columns, where genomic DNA (gDNA) was eliminated and RNA was collected in RNase/DNase free water. RNA samples with an A260/280 ratio of 1.8 to 2.0 were considered pure, and devoid of DNA contamination. Subsequently, RNA samples were analyzed for any changes in transcriptional profile of 84 cell cycle genes by using the Human Cell Cycle RT^2^ *ProfilerTM* PCR Array (Qiagen, Mississauga, Ontario).

As recommended by the manufacturer, extracted mRNA was reverse transcribed to cDNA using the RT^2^ First Strand Kit (Qiagen, Mississauga, Ontario) prior to loading into the QPCR cell cycle array for gene amplification. QPCR conditions were set on the MX3000P® thermocycler (Stratagene, La Jolla, California) according to the manufacturer’s instructions. Gene expression was normalized to five control housekeeping genes [Glyceraldehyde-3-phosphate dehydrogenase (GAPDH), Beta-2-microglobulin (B2M), Hypoxanthine phosphoribosyltransferase 1 (HPRT1), Ribosomal protein L13a (RPL13a), and Beta Actin (ACTB)] using the ΔΔC*_T_* method [10]. To ensure the reliability and quality of the QPCR data, the PCR arrays also contained gDNA control, reverse transcriptase controls and positive PCR controls.

### Trypsinization and Cell Death Experiments

HEK293 cells transfected with EGFP-D2SV or EGFP-C1 control, were trypsinized and reseeded at 30,000 cells in 35 mm dishes with etched grid coverslips (Belko, Vine-land, New Jersey) in accordance with live cell imaging methods, as published by our lab [11]. At five hours post plating, the location of 7–20 transfected cells was recorded. Subsequently, cell adherence was recorded at 20, 48, 68 and 80 hours via fluorescence imaging of live cells with a Leica DMIL inverted microscope, fitted with a DFC500 camera.

### Electron Microscopy and Sample Preparation

HEK293 transfected with cycD2SVmyc were fixed overnight with 4% paraformaldehyde and 0.5% glutaraldehyde in 0.1 M sodium cacodylate buffer. Cells were scraped and collected in eppendorf tubes, and dehydrated in a graded series of ethanol. Cells were embedded in LR White resin (Canemco-Marivac) and sectioned in ultrathin 80 nm slices. The sections were placed on nickel grids where they were washed in sodium borohydride followed by 30 mM glycine in 0.1 M borate buffer (pH 9.6). Sections were then blocked in blotting buffer (5% skimmed milk powder, 3% BSA, 0.1% Tween 20 in PBS) for 45 minutes, incubation with primary cycD2SV antibodies for one hour, followed by secondary anti-rabbit IgG antibodies coupled to 10 nm gold particles (Sigma-Aldrich, Oakville, Ontario) for one hour. After primary and secondary antibody incubations, the sections were washed three times with PBS (five minutes for each wash). Finally, sections were post-fixed in 2.5% glutaraldehyde, washed in PBS, and counterstained with uranyl acetate and lead citrate. For controls, primary cycD2SV antibodies were omitted. Method was adapted based on previous work done in our lab [12].

### Iterative Threading Assembly Refinement (I-TASSER) 3D Protein Structure Prediction Engine

Three-dimensional (3D) protein structure predictions for cycD1, cycD2, cycD3 and cycD2SV were generated by the I-TASSER 3D protein structure prediction engine [13], [14]. The C-score provided is a confidence score used to estimate the quality of the predicted models and usually lies between -5 and 2. A higher C-score value provides a greater model confidence and C-score values greater than -1.5 have a higher confidence in protein folding. Based on these parameters, the cycD2SV ribbon structure most likely represents the correct folding of the protein.

### Statistical Analysis

Unless otherwise stated, all data comparisons were completed using an unpaired two-tail t-test, or a one-way analysis of variance (ANOVA). Significance obtained by ANOVA was further subjected to a Tukey-Kramer’s test for *post-hoc* analysis. Data is expressed as mean ± SEM and was considered statistically significant when the difference in mean values between groups had a P value of 0.05 or less.

## Results

### Overexpression of CycD2SV Promotes Intracellular Protein Aggregation in Immortalized Cell Lines

**Figure 1:**
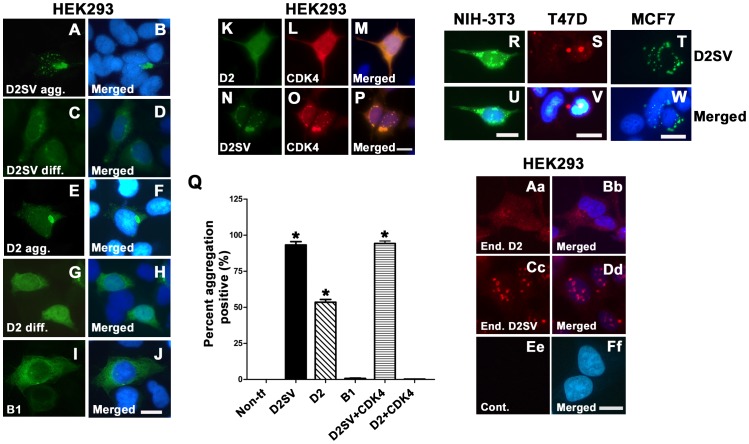
Characterization of cycD2SV aggregation in immortalized cell lines. HEK293 cells transfected with cycD2SVmyc (A–D), cycD2myc (E–H) and cycB1 (I, J) processed for myc (A, C, E, G) and cycB1 (I) immunostaining and nuclear stain (B, D, F, H, J). HEK293 cells co-transfected with cycD2myc and CDK4 (K–M) and cycD2SVmyc and CDK4 (N–P) processed for cycD2SV (K), cycD2 (N) and CDK4 (L, O). NIH-3T3 (R, U), T47D (S, V) and MCF7 (T, W) cells transfected with cycD2SVmyc and processed for myc (R, S, T) immunostaining and nuclear stain (U, V, W). The percentage of HEK293 cells positive for protein aggregation was determined for cycD2SVmyc, cycD2myc, cycB1, cycD2SV plus CDK4 and cycD2 plus CDK4 transfected cells (Q). Cells positive for protein aggregates were quantified and expressed as a percent of total counted cells (Q). Non-transfected (Non-tf) cells stained with myc antibodies were used as a control for protein aggregation. Values are expressed as mean ± SEM. One way ANOVA, *p<0.05 compared to non-transfected control, approximately 1000 cells were counted for each group from three independent experiments (N = 3). Endogenous cycD2 (Aa, Bb) and cycD2SV (Cc, Dd) expression was analyzed in untransfected HEK283 cells. Cells were processed for cycD2 (Aa) and cycD2SV (Cc) immunostaining and nuclear stain (Bb, Dd, and Ff). Primary antibody was omitted as a control (Ee, Ff). Scale bar is 20 µm (A–J; K–P; R, U; S, V; T, W; Aa–Ff).

We have previously demonstrated the ability of cycD2SV to form aggregates and induce cell cycle exit in mouse embryonic cardiomyocytes [4]. Based on this observation we sought to investigate the effects of cycD2SV expression in transformed cell lines. Transfection of both myc tagged and untagged, (data not shown) cycD2SV in HEK293, NIH-3T3, T47D and MCF-7 cell lines revealed a distinct micro-aggregated staining pattern (Fig. 1A, B; R–W) however, there was a small percentage of cells which exhibited a diffuse staining pattern (Fig. 1C, D). In-depth quantitative analysis in HEK293 cells revealed that protein aggregates were present in >90% of HEK293 cells transfected with cycD2SVmyc (Fig. 1Q). In contrast, such protein aggregates were undetectable in control cultures not transfected with any plasmid DNA or cultures transfected with cycB1 (Fig. 1I, J and Q). Subcellular localization studies revealed that cycD2SV protein aggregates were localized exclusively in the cytoplasm or nuclear compartments in approximately 75% of transfected cells, whereas 25% of transfected cells contained protein aggregates in both cytoplasmic and nuclear compartments (data not shown). Interestingly, >50% of cells transfected with cycD2 also revealed protein aggregation in these experiments (Fig. 1E, F and Q) in addition to diffused staining (Fig. 1G, H). In contrast to multiple micro-aggregates in cycD2SV transfected cells, CycD2 overexpressing cells frequently contained one large aggregate which localized to nuclear or perinuclear compartments (Fig. 1E, F). Further, co-expression of CDK4 completely abolished cycD2 but not cycD2SV protein aggregation (Fig. 1K–Q). We also observed a similar micro-aggregation pattern of endogenous cycD2SV protein in non-transfected HEK293 cells using previously described and well characterized polyclonal antibodies [[4], Fig. 1Cc, Dd]. In contrast, immunostaining for endogenous cycD2 in HEK293 cells revealed diffused staining in both nuclear and cytoplasmic compartments, and was rarely associated with aggregate formation (Fig. 1Aa, Bb). Collectively, our results suggest that both endogenous and overexpressed cycD2SV can form micro-aggregates, whereas only overexpressed cycD2 is subjected for aggregation in immortalized cell lines.

### CycD2SV Mediates Cell Cycle Exit in Various Cell Lines

**Figure 2:**
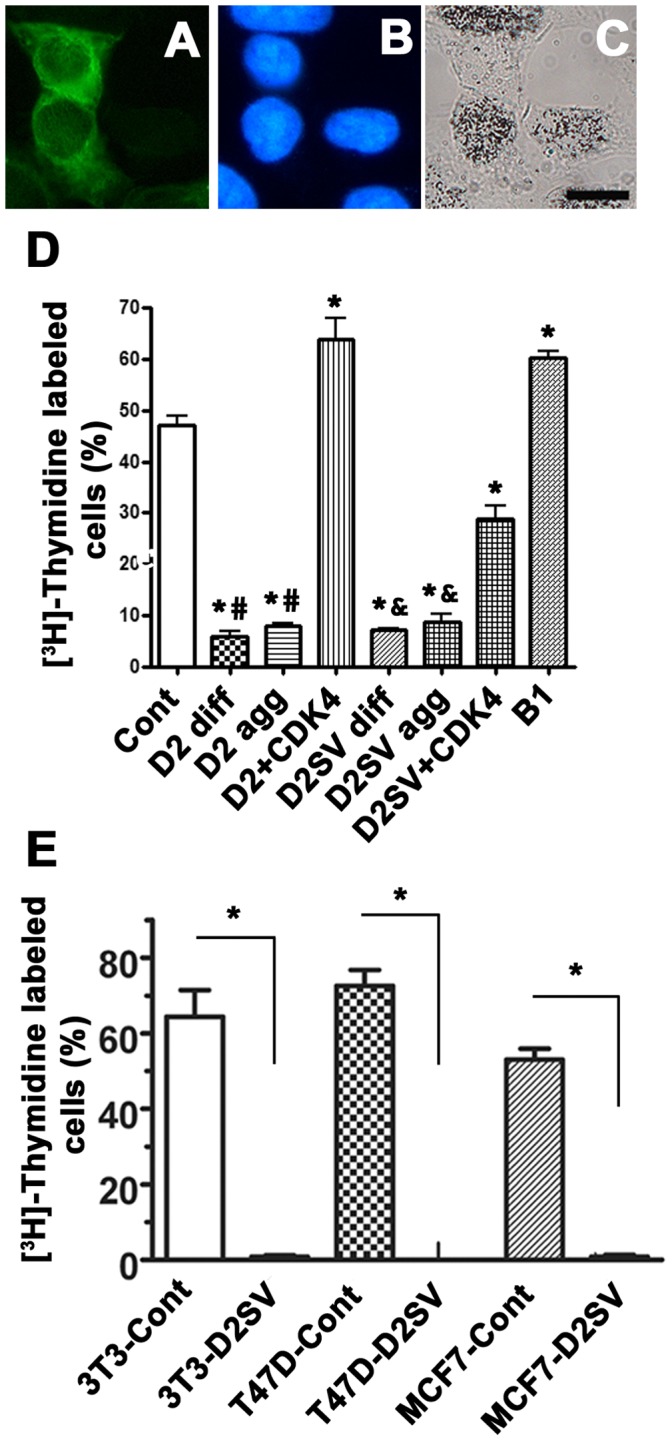
CycD2SV expression decreases the number of cells entering S-phase in immortalized cell lines. Photomicrographs depict examples of [^3^H]-thymidine labelling assay (A–C). Scale bar is 20 µm. HEK293 cells transfected with cycB1 and labeled with [^3^H]-thymidine were visualized by cycB1 immunostaining (A), nuclear stain (B) and [^3^H]-thymidine autoradiography (C). Cells positive for [^3^H]-thymidine contained nuclear silver grains in the nucleus and were visualized under bright field microscopy (C). HEK293 cells transfected with cycB1, cycD2 or cycD2SV were quantified and [^3^H]-thymidine positive cells were expressed as a percent of total counted cells (D). [^3^H]-thymidine counts for cells transfected with cycD2 and cycD2SV were completed for cells with diffused staining (diff.) and those containing protein aggregates (agg.). [^3^H]-thymidine labeling index was also completed for 3T3-NIH, T47D and MCF7 cells overexpressing cycD2SV (E). pcDNA 3.1 vector transfected cells were used for control (cont). Additionally, [^3^H]-thymidine labeling index was completed for cycD2 and cycD2SV cells co-transfected with CDK4. Cells were pulsed with [^3^H]-thymidine for 6hrs (D) or 24 hrs (E). Values are expressed as mean ± SEM. For panel D, statistical analysis was performed using one-way ANOVA; *p<0.05 compared to the LI of control cells, #p<0.05 compared to the LI of cycD2 co-transfected with CDK4 and &p<0.05 compared to the LI of cycD2SV co-transfected with CDK4. For panel E, analysis was performed using unpaired two-tail t-test, *p<0.05 compared to the LI of control cells, approximately 1000 cells were counted for each group from three independent experiments (N = 3).

The major focus of this study was to determine whether cycD2SV expression in non-cardiac cell types leads to a cell cycle arrest similar to the result that we reported for cardiomyocytes, [4] or causes a proliferative phenotype as suggested by an independent research group [3]. To characterize the effects of cycD2SV on cell cycle activity, we first monitored G1/S-phase transit in HEK293 cells transfected with cycD2SV, cycD2, cycB1 or a pcDNA 3.1 vector control. Cells were processed for anti-myc immunostaining and *in situ* [^3^H]-thymidine autoradiography. The labeling index (LI) was assessed as the proportion of the total number of transfected cells that displayed nuclear [^3^H]-thymidine silver grains (Fig. 2A–C). Cells transfected with cycD2SV and cycD2 were identified by myc staining, whereas cells expressing cycB1 were identified by cycB1 antibody staining. In the case of pcDNA 3.1 vector transfected control cultures, we monitored the LI using Hoechst 33342 nuclear staining and silver grains. The LI of HEK293 cells overexpressing cycD2SV was significantly lower when compared to that of non-transfected cells or those transfected with vector alone (8% Vs 49%; approximately six-fold reduction, Fig. 2D). The LIs of cells transfected with cycD2 and cycB1 were also monitored as controls to eliminate the possibility that the observed effects on the cell cycle is due to the general overexpression of cell cycle proteins. While cell cycle activity was significantly elevated in cells expressing cycB1 (approximately 1.3-fold), the G1/S transit rate was significantly decreased in cells expressing cycD2 (approximately six-fold; Fig. 2D). To determine whether protein aggregation affected cell cycle inhibition, [^3^H]-thymidine counts were completed for cycD2 and cycD2SV transfected cells containing protein aggregation or exhibiting diffuse staining. Interestingly both cell populations exhibited similar levels of cell cycle inhibition suggesting that this phenotype is independent of protein aggregation in cells overexpressing cycD2 or cycD2SV (Fig. 2D). Decreasing plasmid concentrations to half during transfections also led to persistent decreases in thymidine labeling in cells expressing cyclin D2 or cycD2SV (data not shown). Additionally, co-transfection of CDK4 with cycD2 abolished cycD2 aggregation (Fig. 1K–M), alleviated cell cycle inhibition and increased cell cycle activity similar to the levels observed in cycB1 transfected cells (Fig. 2D). While co-transfection of CDK4 with cycD2SV increased cell cycle activity by 3 fold compared to single cycD2SV transfected cells, cell cycle activity was not restored to levels observed in control cells (Fig. 2D). Similar to HEK293, other cell lines (NIH-3T3, T47D and MCF-7) transfected with cycD2SV also showed a significant reduction in [^3^H]-thymidine labeling when compared to control transfected cells (Approximately 60 to 80-fold, Fig. 2E). These results suggest that overexpression of cycD2SV in non-cardiac cell types also leads to a cell cycle arrest, but not a proliferative phenotype as suggested by an earlier study [3].

### The 54–136 Amino Acids Region of CycD2SV is Responsible for Cell Cycle Inhibition

**Figure 3:**
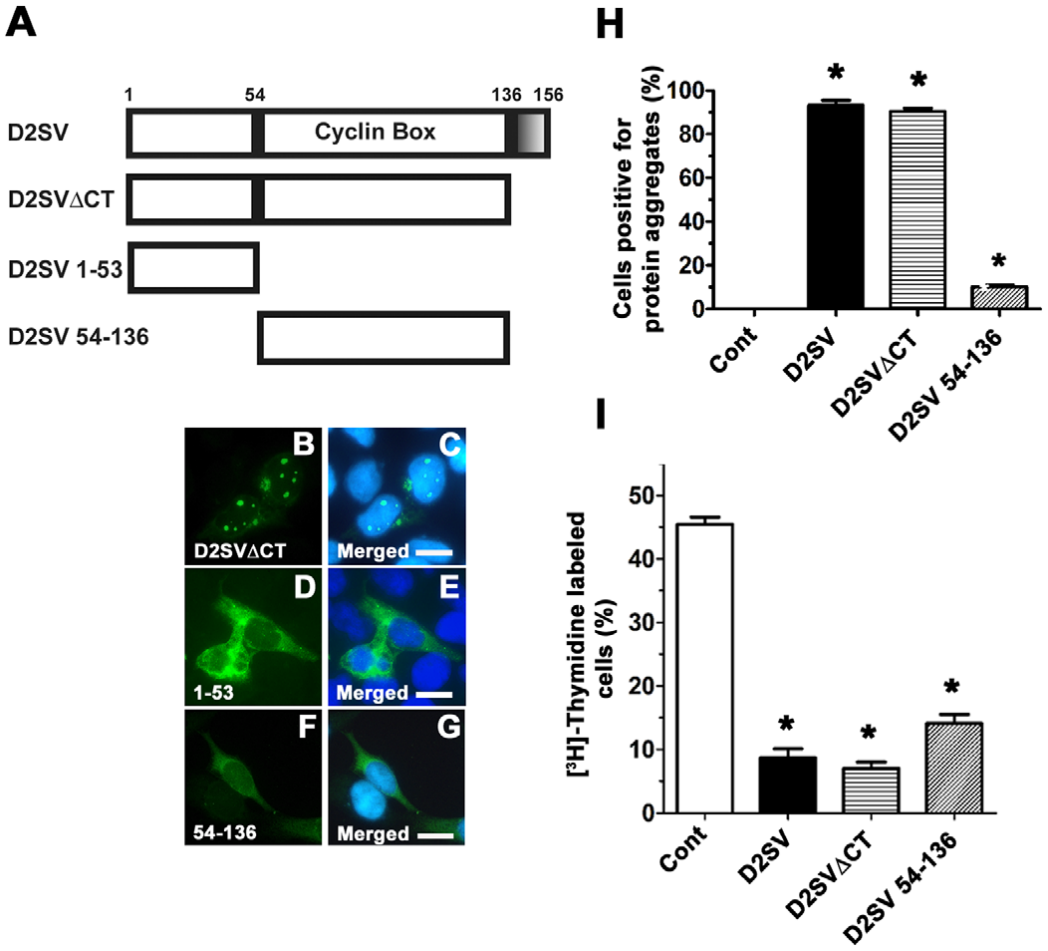
Effects of cycD2SV 54–136 and cycD2SVΔCT overexpression on cell cycle regulation. A schematic representation of D2SVΔCT, D2SV 1–53 and D2SV 54–136 deletions in comparison to full length D2SV (A). Shaded box (136–156 amino acids) in cycD2SV represents the unique CT sequence. HEK293 cells transfected with D2SVΔCTmyc (B, C), D2SV 1–53 (D, E) and D2SV 54–136 myc (F, G) were processed for myc (B, D, F) immunostaining and nuclear stain (C, E, G). Scale bar is 20 µm. HEK293 cells transfected with D2SV, D2SVΔCT and D2SV 54–136 were labeled with [^3^H]-thymidine and processed for immunostaining and [^3^H]-thymidine autoradiography. The percentage of cells positive for protein aggregation (H) and [^3^H]-thymidine (I) were quantified and expressed as a percent of total transfected cells. Cells transfected with pcDNA 3.1 vector were used as a control (cont). Values are expressed as mean ± SEM. One way ANOVA, *p<0.05 compared to control, approximately 1000 cells were counted for each group from three independent experiments (N = 3).

To map the sequence domain(s) responsible for cell cycle inhibition and or protein aggregation, we generated three constructs: cycD2SVΔCT, cycD2SV1-53 and cycD2SV54-136, which code for truncated versions of cycD2SV (Fig. 3A). The cycD2SVΔCT construct encodes the first 136 amino acids common to both cycD2SV and cycD2, but lacks the 20 amino acid CT-region unique to cycD2SV. In contrast, the cycD2SV1-53 construct encodes for amino acids 1–53 and cycD2SV54-136 construct codes for amino acids 54–136 corresponding to the cyclin box in both cycD2 and cycD2SV proteins. The majority of HEK293 cells transfected with cycD2SVΔCT (approximately 90%) contained aggregates, whereas only 10% of cycD2SV54-136 transfected cells contained any aggregation (Fig. 3B, C, F–H). It was also evident that cycD2SVΔCT protein predominantly localized to the nucleus in transfected cells, (Fig. 3B, C) while cycD2SV54-136 immunostaining was diffuse across both the nuclear and cytoplasmic compartments (Fig. 3F, G). Although cells expressing the cycD2SV1-53 construct demonstrated a diffuse staining pattern, they still retained some aggregated masses in the perinuclear area. However the number of cells expressing D2SV1-53 signal was very low (<1% compared to 50–60% of cells positive for other constructs) either due to the unstable nature of the protein or due to a cytotoxic response. (Fig. 3 D, E). The [^3^H]-thymidine incorporation was significantly reduced in cycD2SVΔCT transfected cells, similar to the levels observed in cycD2SV transfected cells (Fig. 3I). Similarly, the LI for cells transfected with cycD2SV54-136 was significantly lower (approximately 3-fold) than that observed in control cells, but was significantly higher (approximately 2-fold) than that observed in cycD2SVΔCT transfected cells (Fig. 3I). Based on these observations, deletion of the unique CT and or 1–53 regions of cycD2SV did not eliminate the cell cycle inhibitory function of cycD2SV protein. However, removal of both 1–53 and the CT region was sufficient to significantly decrease cycD2SV micro-aggregation staining pattern. Collectively, these results suggest that cycD2SV cell cycle inhibitory domain resides in the 54–136 amino acid region, while the 1–53 region may play a major role in cycD2SV aggregation.

### CycD2SV Aggregates Participate in Protein-Protein Interactions with CycD2 and CDK4 in HEK293 Cells

**Figure 4:**
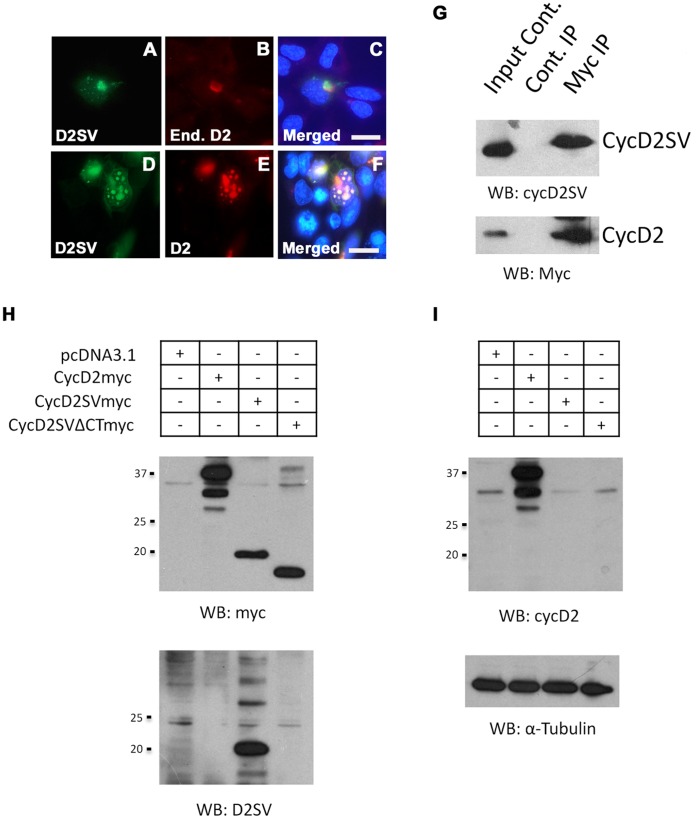
Interaction of cycD2SV with cyclin D2. Overexpressed cycD2SV co-localizes with endogenous and co-transfected cycD2. HEK293 cells transfected with cycD2SVmyc alone (A–C) or co-transfected with cycD2myc (D–F) were processed for myc (A, E), D2SV (D), cycD2 (B) immunostaining and nuclear stain (C, F). Scale bar is 20 µm. Immunoprecipitation analysis of interactions between cycD2SV and cycD2 (G). Western blot (WB) analysis performed on HEK293 cells transfected with pcDNA, cycD2myc, cycD2SVmyc and cycD2SVΔCTmyc using myc and D2SV antibodies (H) as well as cycD2 and α-tubulin antibodies (I). Results in panels H and I indicate the specificity of cycD2SV and cycD2 antibodies and rule out any cross-reactivity.

**Figure 5:**
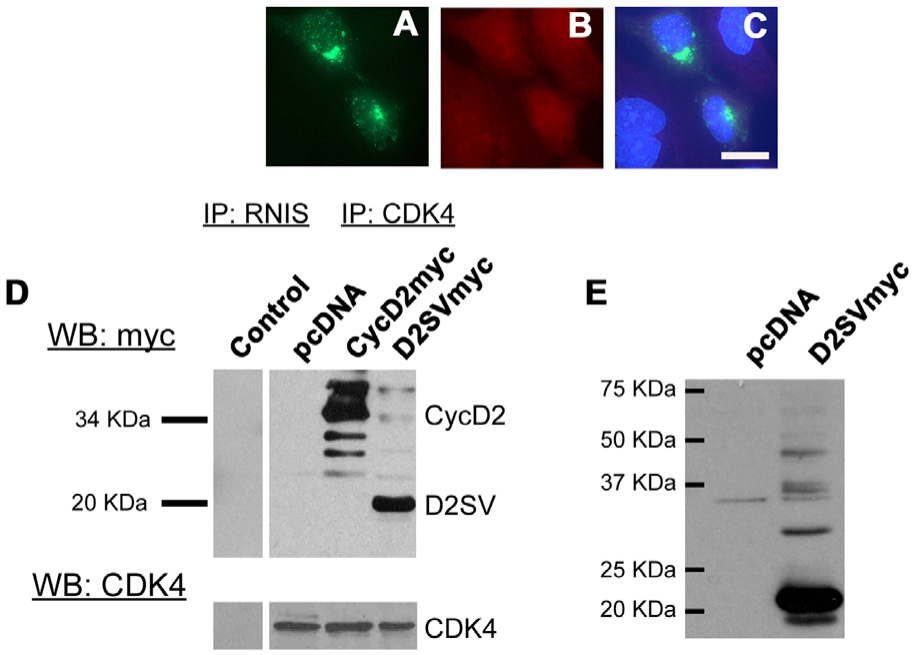
Interaction of cycD2SV with CDK4. HEK293 cells transfected with cycD2SVmyc (A–C) processed for myc (A), CDK4 (B) immunostaining and nuclear stain (C). Interaction of transfected cycD2SV with endogenous CDK4 was determined by CDK4 immunoprecipitation (D). Endogenous CDK4 was immunoprecipitated from pcDNA (negative control), cycD2myc (positive control) and D2SVmyc transfected cells using CDK4 antibodies. An additional immunoprecipitation negative control was completed using rabbit non-immune serum (RNIS, control) on cycD2SVmyc transfected HEK293 cells. Immunoprecipitated samples were resolved by western blot and the nitrocellulose blot was probed with myc and CDK4 antibodies. Western blot analysis of HEK293 cells transfected with pcDNA (control), empty vector, and D2SVmyc using cycD2SV antibodies (E). IP, immunoprecipitation; WB, western blot; End., endogenous.

The 54–136 amino acid region of the cycD2SV contains the majority of the cyclin box and binding sequences for CDK4 and p21^Cip1^ [4], [15]. It is also possible that cycD2SV mediates cell cycle exit in immortalized cell lines by sequestering key cell cycle proteins into aggresomes. Here, we investigated the ability of cycD2SV to physically associate with cycD2 and CDK4 in HEK293 cells using immunostaining and co-IP techniques. Immunostaining experiments demonstrated co-localization of cycD2SV with endogenous cycD2 in approximately 3–5% of cells transfected with cycD2SV (Fig. 4A–C). In contrast, such co-localization was observed in 100% of cells co-transfected with both cycD2SV and cycD2 constructs (Fig. 4D–F). To confirm whether cycD2SV interacts with cycD2, HEK293 cells were co-transfected with myc-tagged cycD2, and untagged cycD2SV constructs. Protein lysates were immunoprecipitated with myc antibodies and immune complexes were collected using protein A-Sepharose beads. IP and supernatant fractions were subjected to western blot analysis using cycD2SV and D2 antibodies and results indicated that cycD2SV forms a complex with cycD2 (Fig. 4G). To ensure cycD2SV antibodies do not cross-react with cycD2 or vice versa, additional western blotting experiments were performed on lysates from cells transfected with myc-tagged cycD2, cycD2SV and cycD2SVΔCT constructs (Fig. 4H, I). In these experiments, cycD2SV antibodies specifically reacted with cycD2SV, but not with cycD2 or cycD2SVΔCT proteins (Fig. 4H), while cycD2 antibodies exclusively reacted with cycD2, but not with cycD2SV or cycD2SVΔCT proteins (Fig. 4I). While immunostaining experiments did not reveal any co-localization of cycD2SV and endogenous CDK4 in transfected HE293 cells (Fig. 5A–C), IP/western analysis of transfected cells with CDK4 and myc antibodies clearly revealed physical interactions between CDK4 and cycD2SV or cycD2 (Fig. 5D). These results confirm the ability of cycD2SV to bind with both cycD2 and CDK4 in non-cardiac cell types.

### CycD2SV Expression Leads to an Impaired ER Stress Associated Protein Degradation and Accumulation of Polyubiquitin Conjugates

**Figure 6:**
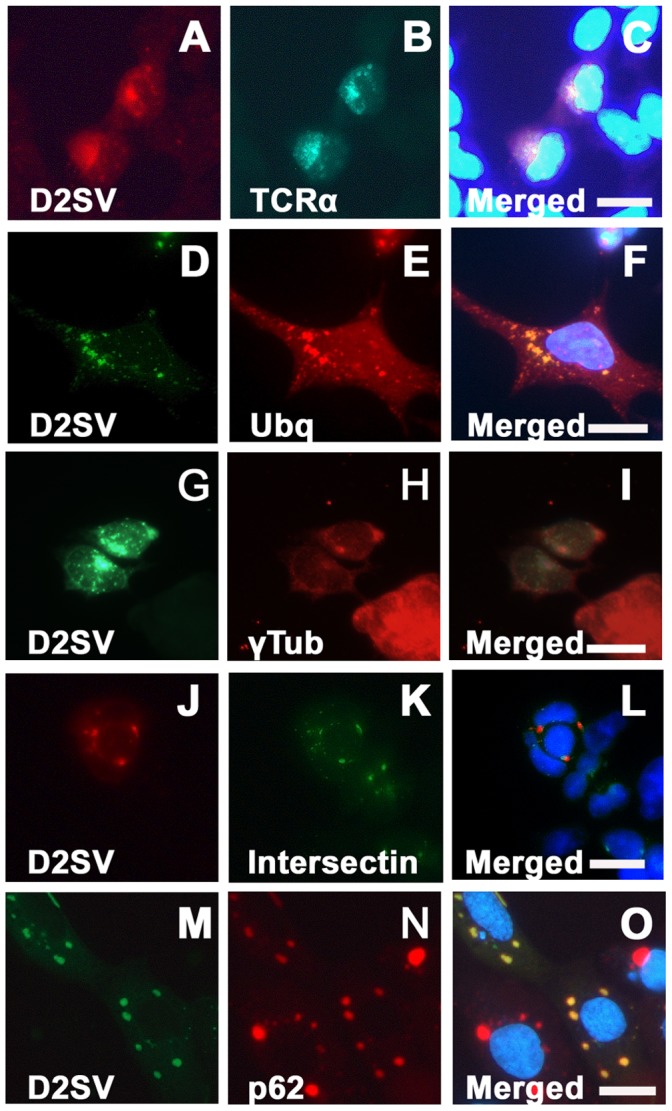
Co-localization of transfected cycD2SV with markers of ER stress and autophagy. HEK293 cells co-transfected with cycD2SVmyc and TCRα-GFP (A–C), cycD2SVmyc and ubiquitin (D–F), cycD2SVmyc and YFP-intersectin (J–L) and cycD2SV-EGFP and mcherry-p62 (M–O) were processed for myc (A, D, G), HA (E) immunostaining and nuclear stain (C, F, I, L). Co-localization of singly transected cycD2SV aggregates in γ-tubulin positive MTOC (G–I). HEK293 cells transfected with cycD2SVmyc (G–I) processed for myc (G), γ-tubulin (H) immunostaining and nuclear stain (E). Scale bar is 20 µm.

Although the predicted molecular weight of mouse cycD2SV is approximately 20 kDa, it has been shown to exist in varying sizes ranging from 20 kDa to >45 kDa in transfected HEK293 cells [[4], Fig. 5E] as well as tissue lysates from postnatal cerebellum [16] or whole embryo extracts [4] under denaturing or non-denaturing conditions. However, the precise nature of these high molecular weight immunoreactive cycD2SV bands is not clear. Intracellular accumulation of misfolded proteins has been shown to trigger protein aggregation and subsequent increases in ubiquitin conjugates as a result of impaired ERAD in neurodegenerative model systems [17]. Next, we examined whether accumulation of cycD2SV aggregates in immortalized cell lines is due to an impairment of ERAD by using a well characterized TCRα-GFP reporter gene system [5]. The misfolded TCRα-GFP reporter was readily eliminated in single transfected HEK293 cells as demonstrated by background GFP fluorescence after 24 hrs (data no shown). In contrast, the GFP fluorescence was retained at higher levels in cells co-transfected with TCRα-GFP and cycD2SV, suggesting an impaired ERAD response in cycD2SV expressing cells (Fig. 6A–C).

To examine ubiquitination profiles, HEK293 cells were co-transfected with cycD2SV and HA tagged ubiquitin (HA-Ubq) constructs and processed for double immunostaining. Co-localization of both proteins in all transfected cells suggests that cycD2SV aggregates are ubiquitinated (100%, Fig. 6D–F). Control cells transfected with HA-Ubq alone showed a diffuse staining with HA antibodies, and did not show any ubiquitinated aggregates similar to co-transfected cells (data not shown). Given that cycD2SV induces ER stress which results in the impairment of ERAD, it is likely that cycD2SV ubq positive aggregates may clog the UPS system. Additionally, γ-tubulin staining, a marker of the microtubule organizing centre (MTOC), co-localized (Fig. 6G–I) with larger aggregates in 28.4% (±4.82 SEM) of cells transfected with cycD2SVmyc. The MTOC has been demonstrated to play a role in the autophagic degradation of protein aggregates [18]. The high levels of γ-tubulin co-localization with cycD2SV aggregates suggest that the rate of cycD2SV aggregation is higher than that of clearance and these aggregates are shuttled to MTOC to minimize cellular toxicity.

### CycD2SV Aggregates are Subjected to Autophagosome Mediated Degradation

**Figure 7:**
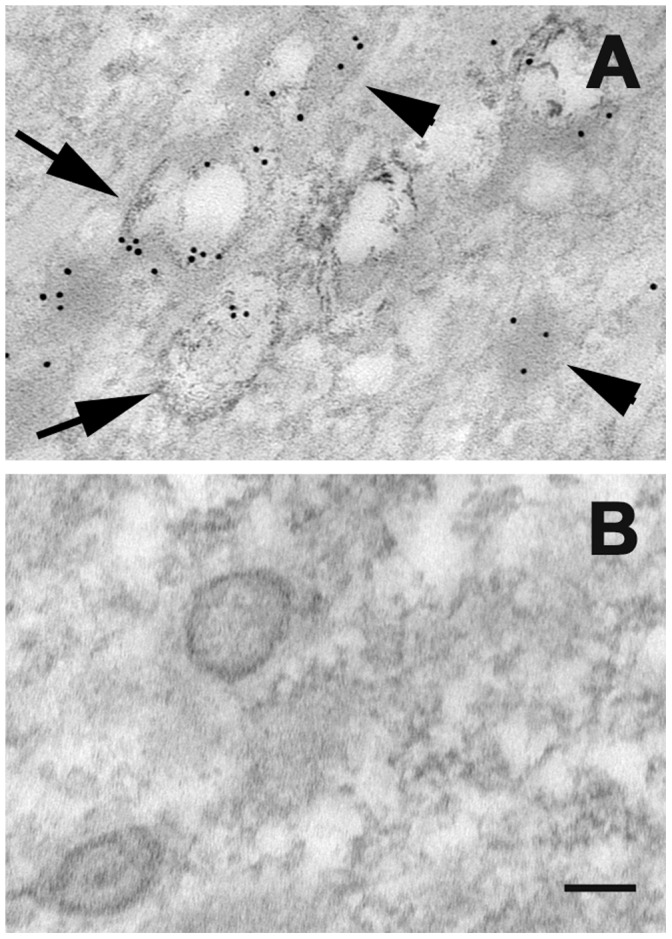
Localization of cycD2SV in electron-dense lysosome (arrowhead) and autophagosome structures (arrows; A, B). HEK293 cells transfected with D2SVmyc (A, B) were fixed, embedded in resin and sectioned (approximately 80 nm). Sections were processed for electron microscopy and probed with cycD2SV antibodies (A). As a control primary antibodies were omitted (B). Scale bar is 100 nm.

Presence of polyubiquitinated aggregates, and impaired ERAD response suggest that alternative protein degradation pathways such as autophagy may be active in cycD2SV expressing cells. Accordingly, cycD2SV expressing cells were further examined using probes specific for critical components in autophagosome formation [19]. In these experiments, cycD2SV aggregates were frequently co-localized with markers specific for early or late endosomes (Intersectin-YFP, Fig. 6J–K) and a selective substrate of autophagy (mCherry-p62, Fig. 6M–O). To obtain direct evidence for the presence of cycD2SV in autophagosome, transfected HEK293 cells were processed for EM analysis using cycD2SV antibodies. Presence of anti-cycD2SV related immunogold particles were readily visible in electron-lucent endosomes (data not shown), electron-dense lysosome and autophagosome structures (Fig. 7A). Such unique immunogold labeling pattern was absent in control sections processed by omitting cycD2SV antibodies (Fig. 7B). Although these ultrastructural analyses suggest that cells overexpressing cycD2SV do not contain large spherical inclusion bodies similar to those reported for the overexpressed mutant Huntingtin protein [20], additional studies are required to further characterize the biophysical nature of the immunoreactive cycD2SV aggregates present within various subcellular structures. Collectively, these results suggest that ubiquitin positive cycD2SV aggregates (Fig. 6D–F) can sequester cell cycle proteins such as cycD2 and CDK4 (Fig. 4A–G, Fig. 5D) and possibly target them for autophagosome mediated degradation (Fig. 6M–O, Fig. 7A).

### CycD2SV Induced G1/S Cell Cycle Exit May Rely on Transcriptional Changes in G2/M but not G1/S Regulatory Genes

**Figure 8:**
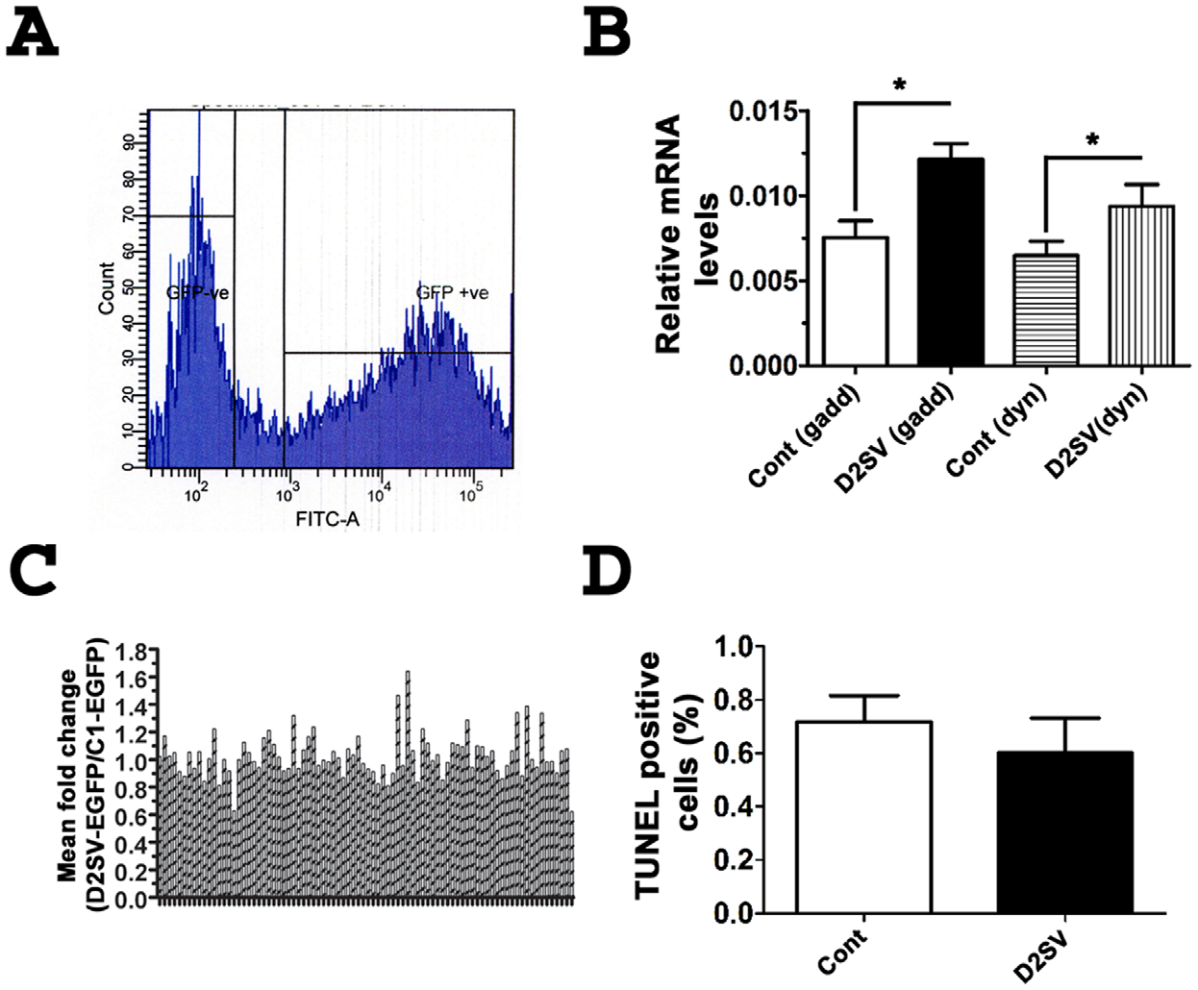
Cell cycle array analysis on cells expressing EGFP-D2SV. Gating for sorting of HEK293 cells transfected with EGFP-D2SV cells during FACS analysis (A). Relative mRNA levels of GADD45α and dynamitin 2 in EGFP-D2SV FACS sorted cells compared to control EGFP-C1 sorted cells (B). A profile of the fold changes of 86 cell cycle genes completed for EGFP-D2SV FACS sorted cells relative to EGFP-C1 control cells (C). Values are expressed as mean ± SEM. Unpaired two-tail t-test, *p<0.05 compared to EGFP-C1, three separate arrays from three independent experiments were analysed (N = 3). HEK293 Cells transfected with cycD2SVmyc were processed for immunostaining using myc antibody followed by TUNNEL staining. TUNNEL positive cells were counted and expressed as a fraction of total cells counted (D). Values are expressed as mean ± SEM. Panel B: Unpaired two-tail t-test, *p<0.05 compared to the LI of untransfected cells, approximately 1000 cells were counted for each group from three independent experiments (N = 3). Panel A: No significant difference was found between groups.

We further sought to determine whether cell cycle exit in cycD2SV expressing cells could result from significant changes in the transcriptional profile of cell cycle genes involved in G1/S and G2/M regulation. For these experiments, total RNA was isolated from FACS sorted cells expressing EGFP or an EGFP-D2SV fusion protein. Total RNA was reverse transcribed, and cDNA samples were subjected to quantitative PCR analysis using human cell cycle QPCR arrays. This high throughput approach enabled us to simultaneously measure fold changes in the transcriptional profile of 86 cell cycle genes (Fig. 8A–C, Table S2). Based on this analysis, there were no significant differences in mRNA levels of the majority of G1/S regulatory genes such as D-type cyclins, CDKs, CKIs and several G1/S check point regulators between control or cycD2SV expressing cells (Table S2). However, we observed a significant increase in the mRNA levels of two G2/M regulatory genes, GADD45α (1.6-fold) and dynamin 2 (1.5-fold, Fig. 8B). GADD45 (growth arrest and DNA-damage inducible protein) α expression is induced by ultraviolet and ionizing radiation, as well as genotoxic stress which subsequently leads to G2/M checkpoint activation, cell cycle arrest, DNA repair, cell survival or apoptosis [21]–[23]. In contrast, dynamin 2 plays a regulatory role in mitosis, cytokinesis, endocytosis and membrane trafficking [24], [25]. Collectively, these results suggest that mRNA levels of the majority of cell cycle genes remain unchanged in cycD2SV expressing cells, as compared to those of control cells. Significant increases in two G2/M related gene transcripts suggest that DNA repair and or mitotic process may be compromised in cells positive for cycD2SV protein aggregation, and these results are consistent with lower [^3^H]-thymidine LI observed in cycD2SV expressing cells.

### Intracellular CycD2SV Protein Aggregation does not Cause Apoptosis in Static Cultures but Sensitizes Cells to Mechanical Stress and Trypsinization Induced Cell Death

**Figure 9:**
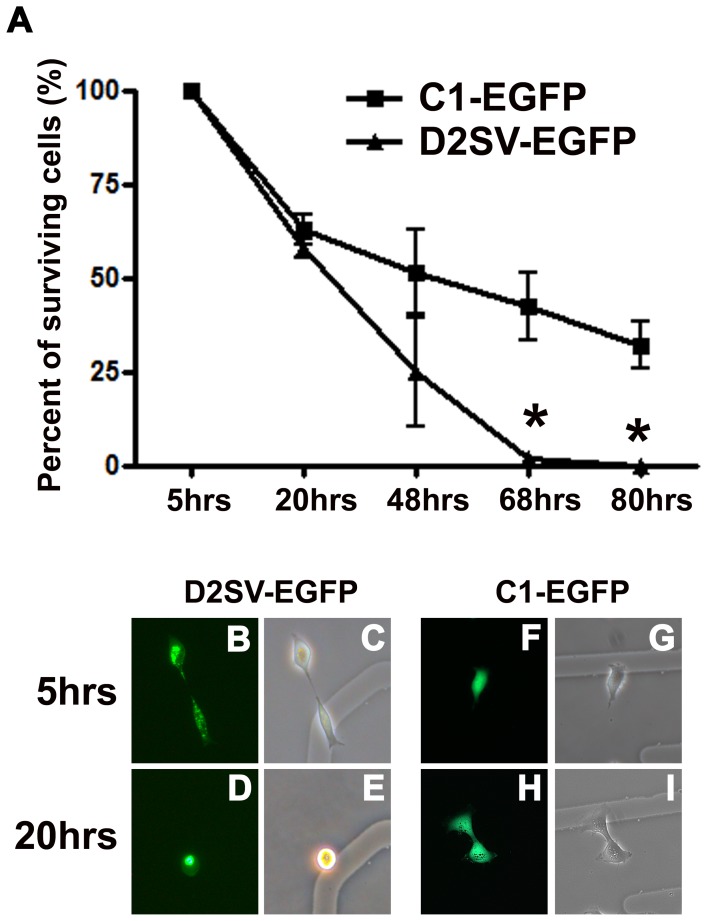
Analysis of D2SV-EGFP induced cell death in collaboration with trypsinization. HEK293 cells transfected with D2SV-EGFP or C1-EGFP were trypsinized and reseeded on gridded coverslips. In each experiment, cells were followed for 80 hrs to determine long term cell viability after trypsinization. Surviving cells were quantified and expressed as a percent of total counted cells (A). Values are expressed as mean ± SEM. One-way ANOVA, *p<0.05, seven to twenty cells were followed from three independent experiments (N = 3). Photomicrographs depict examples of D2SV-EGFP (B–E) and C1-EGFP (F–I) cells at 5 hrs (B, C, F, G) and 20 hrs (D, E, H, I). Note all EGFP-D2SV transfected cells disappeared from culture by 68 hrs post trypsinization (A). Examples of cell death in EGFP-D2SV transfected cells as observed in live cultures by fluorescence microscopy (B–E).

In response to stress stimuli, both GADD45α and Dynamin 2 genes have been shown to cause cell cycle arrest and apoptosis in a p53 dependent manner [22], [26]. Since these two transcripts are upregulated in cycD2SV expressing cells, we assessed the levels of apoptosis using TUNEL and activated Caspase 3 immunostaining assays. Using these methods, no apoptotic cell death was observed in cells transfected with cycD2SV, or a control plasmid (Fig. 8D). Furthermore, cycD2SV expressing cells were viable in subconfluent static cultures for more than a week (data not shown). Apoptotic cells can also be identified during light scatter analysis in a flow cytometer by virtue of significant decreases in the forward scatter, (FSC) and side scatter (SSC) assessments [27]. Consistent with the results observed in static cultures, the number of viable cells on the FSC vs. SSC plots during FACS analysis was similar in cells expressing EGFP or EGFP-D2SV fusion proteins (Fig. S1A, B). Based on a significant decrease in cell cycle levels in cycD2SV expressing cells, we reasoned that FACS sorted EGFP-D2SV cells would have a slower growth potential as compared to EGFP expressing control cells. To further examine this notion, equal numbers of FACS sorted cells were plated in new culture dishes for monitoring growth curves over time. Surprisingly, none of the cycD2SV expressing cells survived over a three day subculture period (Fig. 9A–E). In contrast, FACS sorted cells expressing EGFP survived trypsinization and mechanical stresses imposed by the experimental procedure, albeit initial reductions in cell number (Fig. 9A, F–I). These results suggest that cycD2SV expression may increase cell vulnerability to stress signals.

## Discussion

Although D-type cyclins are overexpressed in many cancers beyond physiological levels, it is clear from the histological analysis that not all cancer cells express similar levels of D-type cyclins within the same tumor sample. To our knowledge, no tumor studies to date have documented whether cancer cells overexpressing cyclin D2 or its splice variants have higher proliferative ability or any detrimental effects on cell cycle progression. Although several cell cycle proteins appear to be dispensable for development, loss of function of many cell cycle proteins appear to specifically affect cell proliferation and differentiation in different tissue types [28]. Similar to cell type specific cell cycle defects observed in genetic loss of function models, sequestration of cyclin D2 and CDK4 in cells overexpressing cycD2SV can also trigger cell cycle defects as reported in this study. Results presented here highlight the fact that cyclin D2 splice variant and its full length counterpart can halt the progression of cell cycle when expressed beyond the normal levels of their binding partner CDK4. Interestingly, coexpression of CDK4 rescues the growth inhibitory effects of cyclin D2 completely but partially blocks the effects of cycD2SV.

Based on the observation that cycD2SV was overexpressed in Graffi retrovirus induced leukemias, Rassart’s group originally hypothesized that this protein could function as an oncogene [2]. Using a mouse embryonic fibroblast (MEF) based focus formation assay, it was shown that cycD2SV failed to induce cellular transformation alone or in combination with c-myc, but was able to induce a large number of foci in combination with activated H-Ras [3]. Although previous studies did not directly examine the role of cycD2SV in G1/S transit control of non-cardiac cell types [3], absence of kinase activity for cycD2SV/CDK4 complex in NIH-3T3 cells [3] and significant decreases in the LIs of various cell lines expressing cycD2SV in this study are in agreement with a growth suppressive role for cycD2SV in cell cycle regulation. The transforming ability of H-Ras in combination with cycD2SV in primary MEFs [3] is particularly intriguing since activated H-Ras failed to transform primary fibroblast cells derived from mouse, rat or human sources [29], [30]. Furthermore, it was shown that immortalization of fibroblasts is a pre-requisite for H-Ras mediated transformation [31].

**Figure 10:**
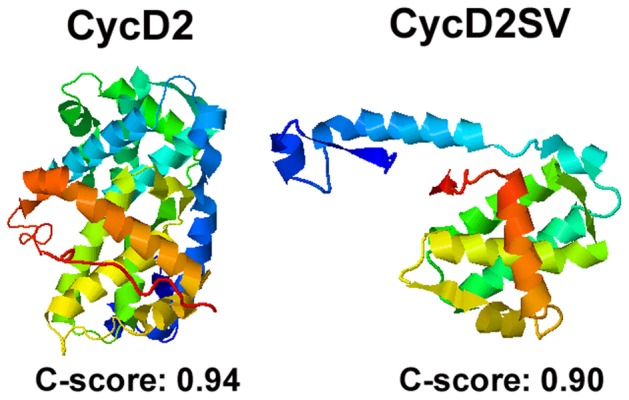
Three-dimensional (3D) protein structure predictions for cycD2 and cycD2SV. Protein structures were determined by the iterative threading assembly refinement (I-TASSER) server, an internet based 3D protein structure prediction engine. The N-terminus of the presented protein structures is denoted by blue and the C-terminus is denoted by red.

The ability of cycD2SV to form micro-aggregates compared to other cyclins such as cycB1 or cycD2 in immortalized cell lines can be directly attributed to a protein misfolding response as evidenced by increased retention of an ERAD reporter [5] in cycD2SV expressing cells (Fig. 6A–C). Generally protein aggregation occurs as a result of misfolding, [32] exposed hydrophobic regions, [33] or insufficient clearance by UPS [18]. Misfolding of cycD2SV could result from its structural divergence, as compared to a number of G1/S or G2/M cyclins. Mammalian cyclins contain two cyclin folds, each comprising five alpha helical structures (Fig. S2). The N-terminal cyclin fold provides a CDK binding interface for all cyclins, while the C-terminal cyclin fold is critical for binding of a CDK activating kinase (CAK) [34], [35]. In contrast to the majority of mammalian cyclins, cycD2SV contains only the first cyclin fold albeit at partial length (54–136 amino acid region). The fifth helical structure (α5) normally found in the CDK4 binding region of D-type cyclins is replaced in cycD2SV with a shorter helix (α5sv) due to insertion of the unique 20 amino acid CT tail (Fig. S2). Crystal structure studies also indicate that stabilization of a given cyclin molecule depends on extensive hydrophobic packaging interactions between different helices in cyclin folds and also those between the NT helical domain with the first three helices of cyclin fold as well as the CT helical domain [34]. Secondary structure analysis revealed that the NT helical domain in cycD2SV is not tightly packed with the cyclin box suggesting a misfolded confirmation (Fig. 10). Since cycD2SV lacks a native structure typically found in other types of cyclins, it is possible that hydrophobic stretches of amino acids (e.g. helix 3) in cycD2SV are exposed unlike other cyclins (Fig. S2, Fig. 10). As a result of this exposure, cellular machinery involved in protein folding may recognize cycD2SV as a partially folded structure.

The ubiquitination pattern of cycD2SV offers an explanation for high molecular weight species of cycD2SV observed in native gel electrophoresis of protein lysates from whole embryo and brain lysates or HEK293 cells [4], [16]. In addition, cycD2SV ubiquitination is somewhat an expected result due to the fact that the stability of endogenous D-type cyclins have been shown to be regulated by UPS degradation [36]–[38]. However, sustained presence of ubiquitin positive aggregates and increased retention of ERAD reporter in cycD2SV expressing cells suggests that overproduction of misfolded proteins may have saturated the ability of UPS system. Consistent with this notion, cycD2SV aggregates were frequently associated with endosomes, lysosomes and autophagosomes, which underscores a critical role for autophagy, a controlled process involving sequestration of certain cytoplasmic contents for lysosomal delivery, where the contents are degraded and recycled [39]. Indeed, association of cycD2SV in p62 positive vesicles further offers a mechanistic explanation for formation of aggresomal inclusions into autophagosomes. The role of p62 in autophagic clearance of polyubiquitinated protein aggregates has been well documented [40], [41]. The carboxy terminal UBA domain of p62 binds to polyubiquitinated proteins, the N-terminal PB1 domain is critical for self-oligomerization and the LRS/LIR domain recruits autophagy regulator LC3/Atg8. It is possible that p62 may link ubiquitinated cycD2SV to the core autophagic protein LC3. However, additional experiments are required to confirm this notion.

In contrast to a high frequency of multiple protein aggregates seen with cycD2SV expression, cycD2 expressing cells frequently contained a single large aggregate in the absence of equal amounts of CDK4 expression. Although exogenously expressed cycD2 aggregates were ubiquitinated, cycD2 expressing cells were not positive for markers of autophagy or ER stress (Chinni and Pasumarthi, unpublished data). Absence of such intense perinuclear staining pattern for endogenous cycD2 in HEK293 cells suggests that alternative mechanisms other than ER stress and or autophagy may be responsible for aggregation of overexpressed cycD2. Misfolding of cycD2 due to fusion with a myc epitope in HEK293 cells can be readily ruled out since exogenous expression of the same cycD2myc fusion protein in embryonic cardiomyocytes did not induce any protein aggregation [4]. It is possible that high intracellular concentrations of cycD2 may promote dimer or oligomer formation. Consistent with this notion, IP studies using differentially tagged cycD2 constructs (cycD2-DsRed and D2myc) revealed the dimerization possibility of cycD2 molecules either via direct or indirect protein-protein interactions (Zhang and Pasumarthi, unpublished data).

Recent studies showed that another cell cycle regulator p57^kip2^ is capable of associating with itself via the NH2 domain to form a homodimeric species which is a more potent inhibitor of the cycD1/CDK4 complex compared to a single p57^kip2^ molecule [42]. However, it is not known whether homodimers of CKIs such as p57^kip2^ can promote dimerization of cycD/CDK4 heterodimers. Dimerization of CDK4 molecules in two adjacent cycD/CDK4 heterodimers similar to that described for kinase domains of EGF receptors [43] may also offer an explanation for perinuclear cycD2 aggregates. Furthermore, crystal structure studies involving cycD3/CDK4 complex revealed the existence of two copies of cycD3/CDK4 in each crystal and also suggested that the CT cyclin fold may be responsible for dimerization. The cycD3-cycD3 interactions in crystals were thought to be due to the absence of a structured CT tail in cycD3 similar to that found in cycA, B or E that would be expected to shield the surface of the CT cyclin fold [44]. Secondary structure analysis via I-Tasser [13], [14] also revealed absence of a structured CT tail, distal to the second cyclin fold in cycD2 and cycD1 molecules (Fig. S3, Fig. 10). These structural similarities indeed suggest that cycD2-cycD2 dimerization may be possible through a second cyclin fold similar to that described for cycD3-cycD3 dimerization.

In the present study, we have also shown that cycD2SV which lacks the second cyclin fold can interact with cycD2 by IP or immunolocalization experiments. Interestingly, deletion of 1–53 amino acid NT region of cycD2SV significantly abolished intracellular protein aggregation. This in turn suggests that cycD2-cycD2 or cycD2-cycD2SV dimerization may occur through the structured NT sequence (cycD2SV 1–53) similar to that described for p57^kip2^ [42]. Since cycD2SV 54–136 protein still retains the ability to bind CDK4 (Zhang and Pasumarthi, unpublished data) and exhibits a significant reduction in its tendency for protein aggregation, CDK4-CDK4 interactions may not play a critical role when it comes to cycD2SV aggregation. Collectively, our immunostaining and IP/western studies show for the first time that, under high intracellular concentrations, cycD2 may exist as dimers with cycD2 and or cycD2SV *in vivo*. These results may certainly offer a mechanistic explanation for earlier studies describing cytoplasmic sequestration of endogenous or overexpressed cycD1 in a variety of mammalian cancer cell lines and postmitotic neurons [45], [46]. Further studies are necessary to precisely identify the cellular compartment(s) where the initial sequestrations of cycD2SV, cycD2 and CDK4 molecules occur.

To elucidate the possible mechanism(s) of cycD2SV induced cell cycle exit, we investigated its ability to interact with CDK4 and cycD2 based on the premise that sequestration of these positive cell cycle regulators by cycD2SV which lacks the CAK binding domain would render them inactive. Consistent with this notion, an earlier study demonstrated that immunocomplexes containing cycD2SV and CDK4 failed to phosphorylate pRb, a critical step required for cells to overcome G1/S restriction point [3]. Indeed, our results in the present study showed that cycD2SV can sequester CDK4 and cycD2 into p62 positive inclusion bodies and possibly target them for UPS/autophagy mediated degradation. To our knowledge, this is the first report which shows that a cyclin variant can directly affect the stability of other cyclins and or CDK complexes. Consistent with this notion, we have recently shown that endogenous levels of cycD2SV are upregulated in HEK293 cells subjected to confluence and serum starvation. Further, ablation of cycD2SV protein levels has led to increased levels of cycD2 protein and cells entering S-phase during confluence (Wafa *et al.*, In preparation).

We further present evidence for the upregulation of GADD45α and dynamin 2 transcripts in response to cycD2SV overexpression. GADD45α is a p53 inducible gene which plays a role in cell cycle arrest in response to double stranded DNA damage [47]. Additionally, GADD45α is capable of inducing G2/M arrest by interfering with the formation of cycB/CDK1 complexes [21], [23]. Given the established role of GADD45α, an alternate mechanism by which cycD2SV actuates cell cycle exit might be due to induction of DNA damage. However, based on 53BP1 (p53-binding protein 1) staining, a marker for double stranded DNA breaks, no DNA damage was detected in cycD2SV transfected cells (Data not shown). Overexpression of Dynamin 2 was shown to decrease cell proliferation and cause cell death via p53 pathway [26]. However, cycD2SV positive cells were negative for apoptosis. Currently, the mechanism by which cycD2SV upregulates these proteins is yet to be elucidated. It would be interesting to investigate whether cycD2SV is able to upregulate these transcripts in a p53 dependent or independent manner.

In a study by Huang *et al*., cell trypsinization was demonstrated to upregulate the pro-apoptotic protein p53, the CKI p21 and the down regulation of the pro-survival protein Bcl-2 [48]. Interestingly, cycD2SV transfected cell subjected to trypsinization did not survive in culture 48 hrs post re-plating. While cycD2SV aggregation alone is not enough to induce apoptotic cell death, it is possible that cycD2SV expressing cells are sensitized to cell death upon exposure to additional stressors such as trypsinization. However, additional experimental work is needed to elucidate the mechanism underlying cycD2SV sensitization phenomenon.

Due to a positive regulatory role of D type cyclins in promoting cell cycle progression, aberrant expression of these cyclins have been implicated in various types of cancers [49]. Alternatively, D type cyclins have also been implicated in cellular senescence, a state of G1 arrest where cells no longer respond to mitogenic signals [6], [50], [51]. Research conducted by Pagano *et al.* demonstrated that microinjection of cycD1 into G1 synchronized or UV exposed human lung IMR-90 fibroblasts dramatically decreased cells entering S-phase by preventing nuclear localization of PCNA [52]. PCNA, an auxiliary protein of DNA polymerases δ and ε is required for DNA replication or repair and is known to interact with cycD1 [52], [53]. Pagano *et al* also showed that co-injection of PCNA but not CDK4 or CDK2 expression constructs prevented cycD1 induced replicative arrest. Overexpression of cycD1 was also implicated in cell cycle exit in Hs68 fibroblasts by inactivation of CDK2 kinase activity [54]. In the present study, overexpression of cycD2 in HEK293 cells was associated with a significant decrease in S-phase entry albeit not as effective as cycD2SV. While this result was surprising due to an established role of cycD2 in cell cycle activation, similar to cycD1, cycD2 has also been implicated in cellular senescence as well as various states of growth arrest [6]. Both cycD2 mRNA and protein levels were found to be significantly increased in cells subjected to contact inhibition, serum starvation and cellular senescence. Under these conditions, cycD2 was shown to form inactive complexes with CDK2 by preventing its normal association with obligate binding partners such as cycA or cycE [6]. Collectively, these studies suggest that while D-type cyclins play an important role in cell cycle progression, under certain conditions they may also play a key role in cell cycle exit so as to ensure that cell cycle progression does not occur prematurely.

## Supporting Information

Figure S1 Selection gating for viable cells during FACS sorting of C1-EGFP (A) and D2SV-EGFP (B) transfected cells. Side scatter (SSC) and forward scatter (FSC) plots (A, B) were used to determine predicted viable cells for FACS sorting. Region 1 (R1) contains viable cells where as region 2 (R2) contains cells in early stages of apoptosis and region 3 (R3) contains dead cells. Cell shrinkage and nuclear condensations are two of the hallmarks of apoptosis. Cell shrinkage leads to a decrease in forward scatter (FSC) whereas nuclear condensation results in an increase in side scatter (SSC). EGFP-C1 and EGFP-cycD2SV sorted cells roughly contained same number of cells in regions 2 and 3.
(TIF)

Figure S2 **Sequence alignment of D-type cyclins and cycD2SV reveals important conserved domains.** Cyclins in general contain two important cyclin folds, the N-terminal cyclin fold (red box) and the C-terminal cyclin fold (black box) each containing five alpha-helical domains. For clarity, the NT helical domains are labeled as α1-5 and the CT helical domains are labeled as α1′-5′. In general, cyclins also contain two additional NT and CT helical domains (αNT, αCT) located outside of the two cyclin folds. However, D-type cyclins appear to lack the αCT domain. The NT cyclin fold also known as the cyclin box is responsible for the association of cyclins with CDKs while the CT cyclin fold is thought to be responsible for binding of CDK activating kinase (CAK) and proper folding of the cyclin (GenBank: AAA37519.1). The cycD2SV CT sequence is highlighted in yellow. Helical domains are denoted by blue cylinders. The orange cylinder marks the helical domain (α5sv) present in the cycD2SV unique CT-domain. Asterix (*) denotes amino acids which are identical among all sequences. α denotes α-helix.
(TIF)

Figure S3 **Three-dimensional (3D) protein structure predictions for cycD1 and cycD3.** Protein structures were determined by the iterative threading assembly refinement (I-TASSER) server, an internet based 3D protein structure prediction engine. The N-terminus of the presented protein structures is denoted by blue and the C-terminus is denoted by red.
(TIF)

Table S1 Primers used for generation of DNA expression constructs.
(DOC)

Table S2 Cell cycle array analysis completed for C1-EGFP control and D2SV-EGFP sorted cells.
(DOC)

## References

[1] Johnson DG, Walker CL (1999) Cyclins and cell cycle checkpoints. Annu Rev Pharmacol Toxicol 39: 295–312. 10331086 10.1146/annurev.pharmtox.39.1.295

[2] Denicourt C, Kozak CA, Rassart E (2003) Gris1, a new common integration site in Graffi murine leukemia virus-induced leukemias: overexpression of a truncated cyclin D2 due to alternative splicing. J Virol 77: 37–44. 12477808 10.1128/JVI.77.1.37-44.2003 PMC140601

[3] Denicourt C, Legault P, McNabb FA, Rassart E (2008) Human and mouse cyclin D2 splice variants: transforming activity and subcellular localization. Oncogene 27: 1253–1262. 17873913 10.1038/sj.onc.1210750

[4] Sun Q, Zhang F, Wafa K, Baptist T, Pasumarthi KB (2009) A splice variant of cyclin D2 regulates cardiomyocyte cell cycle through a novel protein aggregation pathway. J Cell Sci 122: 1563–1573. 19401331 10.1242/jcs.047738

[5] DeLaBarre B, Christianson JC, Kopito RR, Brunger AT (2006) Central pore residues mediate the p97/VCP activity required for ERAD. Mol Cell 22: 451–462. 16713576 10.1016/j.molcel.2006.03.036

[6] Meyyappan M, Wong H, Hull C, Riabowol KT (1998) Increased expression of cyclin D2 during multiple states of growth arrest in primary and established cells. Mol Cell Biol 18: 3163–3172. 9584157 10.1128/mcb.18.6.3163 PMC108898

[7] Lamark T, Perander M, Outzen H, Kristiansen K, Overvatn A, et al (2003) Interaction codes within the family of mammalian Phox and Bem1p domain-containing proteins. J Biol Chem 278: 34568–34581. 12813044 10.1074/jbc.M303221200

[8] Pankiv S, Clausen TH, Lamark T, Brech A, Bruun JA, et al (2007) p62/SQSTM1 binds directly to Atg8/LC3 to facilitate degradation of ubiquitinated protein aggregates by autophagy. J Biol Chem 282: 24131–24145. 17580304 10.1074/jbc.M702824200

[9] Mohney RP, Das M, Bivona TG, Hanes R, Adams AG, et al (2003) Intersectin activates Ras but stimulates transcription through an independent pathway involving JNK. J Biol Chem 278: 47038–47045. 12970366 10.1074/jbc.M303895200

[10] Livak KJ, Schmittgen TD (2001) Analysis of relative gene expression data using real-time quantitative PCR and the 2(-Delta Delta C(T)) Method. Methods 25: 402–408. 11846609 10.1006/meth.2001.1262

[11] McMullen NM, Zhang F, Hotchkiss A, Bretzner F, Wilson JM, et al (2009) Functional characterization of cardiac progenitor cells and their derivatives in the embryonic heart post-chamber formation. Dev Dyn 238: 2787–2799. 19842178 10.1002/dvdy.22112

[12] Zhang F, Pasumarthi KB (2007) Ultrastructural and immunocharacterization of undifferentiated myocardial cells in the developing mouse heart. J Cell Mol Med 11: 552–560. 17635645 10.1111/j.1582-4934.2007.00044.x PMC3922360

[13] Roy A, Kucukural A, Zhang Y (2010) I-TASSER: a unified platform for automated protein structure and function prediction. Nat Protoc 5: 725–738. 20360767 10.1038/nprot.2010.5 PMC2849174

[14] Zhang Y (2008) I-TASSER server for protein 3D structure prediction. BMC Bioinformatics 9: 40. 18215316 10.1186/1471-2105-9-40 PMC2245901

[15] Zwicker J, Brusselbach S, Jooss KU, Sewing A, Behn M, et al (1999) Functional domains in cyclin D1: pRb-kinase activity is not essential for transformation. Oncogene 18: 19–25. 9926916 10.1038/sj.onc.1202286

[16] Kajitani K, Wafa K, Pasumarthi KB, Robertson GS (2010) Developmental expression of the cyclin D2 splice variant in postnatal Purkinje cells of the mouse cerebellum. Neurosci Lett 477: 100–104. 20433897 10.1016/j.neulet.2010.04.042

[17] Bence NF, Sampat RM, Kopito RR (2001) Impairment of the ubiquitin-proteasome system by protein aggregation. Science 292: 1552–1555. 11375494 10.1126/science.292.5521.1552

[18] Kirkin V, McEwan DG, Novak I, Dikic I (2009) A role for ubiquitin in selective autophagy. Mol Cell 34: 259–269. 19450525 10.1016/j.molcel.2009.04.026

[19] Razi M, Chan EY, Tooze SA (2009) Early endosomes and endosomal coatomer are required for autophagy. J Cell Biol 185: 305–321. 19364919 10.1083/jcb.200810098 PMC2700373

[20] Waelter S, Boeddrich A, Lurz R, Scherzinger E, Lueder G, et al (2001) Accumulation of mutant huntingtin fragments in aggresome-like inclusion bodies as a result of insufficient protein degradation. Mol Biol Cell 12: 1393–1407. 11359930 10.1091/mbc.12.5.1393 PMC34592

[21] Wang XW, Zhan Q, Coursen JD, Khan MA, Kontny HU, et al (1999) GADD45 induction of a G2/M cell cycle checkpoint. Proc Natl Acad Sci U S A 96: 3706–3711. 10097101 10.1073/pnas.96.7.3706 PMC22358

[22] Liebermann DA, Hoffman B (2007) Gadd45 in the response of hematopoietic cells to genotoxic stress. Blood Cells Mol Dis 39: 329–335. 17659913 10.1016/j.bcmd.2007.06.006 PMC3268059

[23] Zhan Q, Antinore MJ, Wang XW, Carrier F, Smith ML, et al (1999) Association with Cdc2 and inhibition of Cdc2/Cyclin B1 kinase activity by the p53-regulated protein Gadd45. Oncogene 18: 2892–2900. 10362260 10.1038/sj.onc.1202667

[24] Evans DL, Harris DT, Jaso-Friedmann L (1990) Effects of phorbol esters and calcium ionophore on nonspecific cytotoxic cells. Dev Comp Immunol 14: 223–230. 2115013 10.1016/0145-305x(90)90093-t

[25] Praefcke GJ, McMahon HT (2004) The dynamin superfamily: universal membrane tubulation and fission molecules? Nat Rev Mol Cell Biol 5: 133–147. 15040446 10.1038/nrm1313

[26] Fish KN, Schmid SL, Damke H (2000) Evidence that dynamin-2 functions as a signal-transducing GTPase. J Cell Biol 150: 145–154. 10893263 10.1083/jcb.150.1.145 PMC2185575

[27] Williams O (2004) Apoptosis Methods and Protocols; Brady HJM, editor. Totowa, NJ: Humana Press Inc. 31–42 p.

[28] Ciemerych MA, Sicinski P (2005) Cell cycle in mouse development. Oncogene 24: 2877–2898. 15838522 10.1038/sj.onc.1208608

[29] Serrano M, Lin AW, McCurrach ME, Beach D, Lowe SW (1997) Oncogenic ras provokes premature cell senescence associated with accumulation of p53 and p16INK4a. Cell 88: 593–602. 9054499 10.1016/s0092-8674(00)81902-9

[30] Wei S, Sedivy JM (1999) Expression of catalytically active telomerase does not prevent premature senescence caused by overexpression of oncogenic Ha-Ras in normal human fibroblasts. Cancer Res 59: 1539–1543. 10197626

[31] Newbold RF, Overell RW (1983) Fibroblast immortality is a prerequisite for transformation by EJ c-Ha-ras oncogene. Nature 304: 648–651. 6877385 10.1038/304648a0

[32] Garcia-Mata R, Gao YS, Sztul E (2002) Hassles with taking out the garbage: aggravating aggresomes. Traffic 3: 388–396. 12010457 10.1034/j.1600-0854.2002.30602.x

[33] Wedegaertner PB (2002) Characterization of subcellular localization and stability of a splice variant of G alpha i2. BMC Cell Biol 3: 12. 12057015 10.1186/1471-2121-3-12 PMC116600

[34] Brown NR, Noble ME, Endicott JA, Garman EF, Wakatsuki S, et al (1995) The crystal structure of cyclin A. Structure. 3: 1235–1247. 10.1016/s0969-2126(01)00259-3 8591034

[35] Diehl JA, Sherr CJ (1997) A dominant-negative cyclin D1 mutant prevents nuclear import of cyclin-dependent kinase 4 (CDK4) and its phosphorylation by CDK-activating kinase. Mol Cell Biol 17: 7362–7374. 9372967 10.1128/mcb.17.12.7362 PMC232592

[36] Lin DI, Barbash O, Kumar KG, Weber JD, Harper JW, et al (2006) Phosphorylation-dependent ubiquitination of cyclin D1 by the SCF(FBX4-alphaB crystallin) complex. Mol Cell 24: 355–366. 17081987 10.1016/j.molcel.2006.09.007 PMC1702390

[37] Kida A, Kakihana K, Kotani S, Kurosu T, Miura O (2007) Glycogen synthase kinase-3beta and p38 phosphorylate cyclin D2 on Thr280 to trigger its ubiquitin/proteasome-dependent degradation in hematopoietic cells. Oncogene 26: 6630–6640. 17486076 10.1038/sj.onc.1210490

[38] Okabe H, Lee SH, Phuchareon J, Albertson DG, McCormick F, et al (2006) A critical role for FBXW8 and MAPK in cyclin D1 degradation and cancer cell proliferation. PLoS One 1: e128. 17205132 10.1371/journal.pone.0000128 PMC1762433

[39] Klionsky DJ (2010) The autophagy connection. Dev Cell 19: 11–12. 20643346 10.1016/j.devcel.2010.07.005 PMC2915768

[40] Moscat J, Diaz-Meco MT (2009) p62 at the crossroads of autophagy, apoptosis, and cancer. Cell 137: 1001–1004. 19524504 10.1016/j.cell.2009.05.023 PMC3971861

[41] Komatsu M, Ichimura Y (2010) Physiological significance of selective degradation of p62 by autophagy. FEBS Lett 584: 1374–1378. 20153326 10.1016/j.febslet.2010.02.017

[42] Reynaud EG, Guillier M, Leibovitch MP, Leibovitch SA (2000) Dimerization of the amino terminal domain of p57Kip2 inhibits cyclin D1-cdk4 kinase activity. Oncogene 19: 1147–1152. 10713702 10.1038/sj.onc.1203403

[43] Zhang X, Gureasko J, Shen K, Cole PA, Kuriyan J (2006) An allosteric mechanism for activation of the kinase domain of epidermal growth factor receptor. Cell 125: 1137–1149. 16777603 10.1016/j.cell.2006.05.013

[44] Takaki T, Echalier A, Brown NR, Hunt T, Endicott JA, et al (2009) The structure of CDK4/cyclin D3 has implications for models of CDK activation. Proc Natl Acad Sci U S A 106: 4171–4176. 19237555 10.1073/pnas.0809674106 PMC2657433

[45] Sumrejkanchanakij P, Tamamori-Adachi M, Matsunaga Y, Eto K, Ikeda MA (2003) Role of cyclin D1 cytoplasmic sequestration in the survival of postmitotic neurons. Oncogene 22: 8723–8730. 14647467 10.1038/sj.onc.1206870

[46] Alao JP, Gamble SC, Stavropoulou AV, Pomeranz KM, Lam EW, et al (2006) The cyclin D1 proto-oncogene is sequestered in the cytoplasm of mammalian cancer cell lines. Mol Cancer 5: 7. 16503970 10.1186/1476-4598-5-7 PMC1388232

[47] Hollander MC, Fornace AJ Jr (2002) Genomic instability, centrosome amplification, cell cycle checkpoints and Gadd45a. Oncogene 21: 6228–6233. 12214253 10.1038/sj.onc.1205774

[48] Huang HL, Hsing HW, Lai TC, Chen YW, Lee TR, et al (2010) Trypsin-induced proteome alteration during cell subculture in mammalian cells. J Biomed Sci 17: 36. 20459778 10.1186/1423-0127-17-36 PMC2873939

[49] von Bergwelt-Baildon MS, Kondo E, Klein-Gonzalez N, Wendtner CM (2011) The cyclins: a family of widely expressed tumor antigens? Expert Rev Vaccines 10: 389–395. 21434806 10.1586/erv.10.170

[50] Han EK, Ng SC, Arber N, Begemann M, Weinstein IB (1999) Roles of cyclin D1 and related genes in growth inhibition, senescence and apoptosis. Apoptosis 4: 213–219. 14634283 10.1023/a:1009618824145

[51] Garkavtsev I, Hull C, Riabowol K (1998) Molecular aspects of the relationship between cancer and aging: tumor suppressor activity during cellular senescence. Exp Gerontol 33: 81–94. 9467719 10.1016/s0531-5565(97)00086-7

[52] Pagano M, Theodoras AM, Tam SW, Draetta GF (1994) Cyclin D1-mediated inhibition of repair and replicative DNA synthesis in human fibroblasts. Genes Dev 8: 1627–1639. 7958844 10.1101/gad.8.14.1627

[53] Matsuoka S, Yamaguchi M, Matsukage A (1994) D-type cyclin-binding regions of proliferating cell nuclear antigen. J Biol Chem 269: 11030–11036. 7908906

[54] Atadja P, Wong H, Veillete C, Riabowol K (1995) Overexpression of cyclin D1 blocks proliferation of normal diploid fibroblasts. Exp Cell Res 217: 205–216. 7698220 10.1006/excr.1995.1080

