# Supplementary figures and images for: Characterization of Growth Suppressive Functions of a Splice Variant of Cyclin D2
Source: PLoS One. 2013 Jan 10;8(1):e53503. doi: 10.1371/journal.pone.0053503 (PMC3542336; doi:10.1371/journal.pone.0053503)

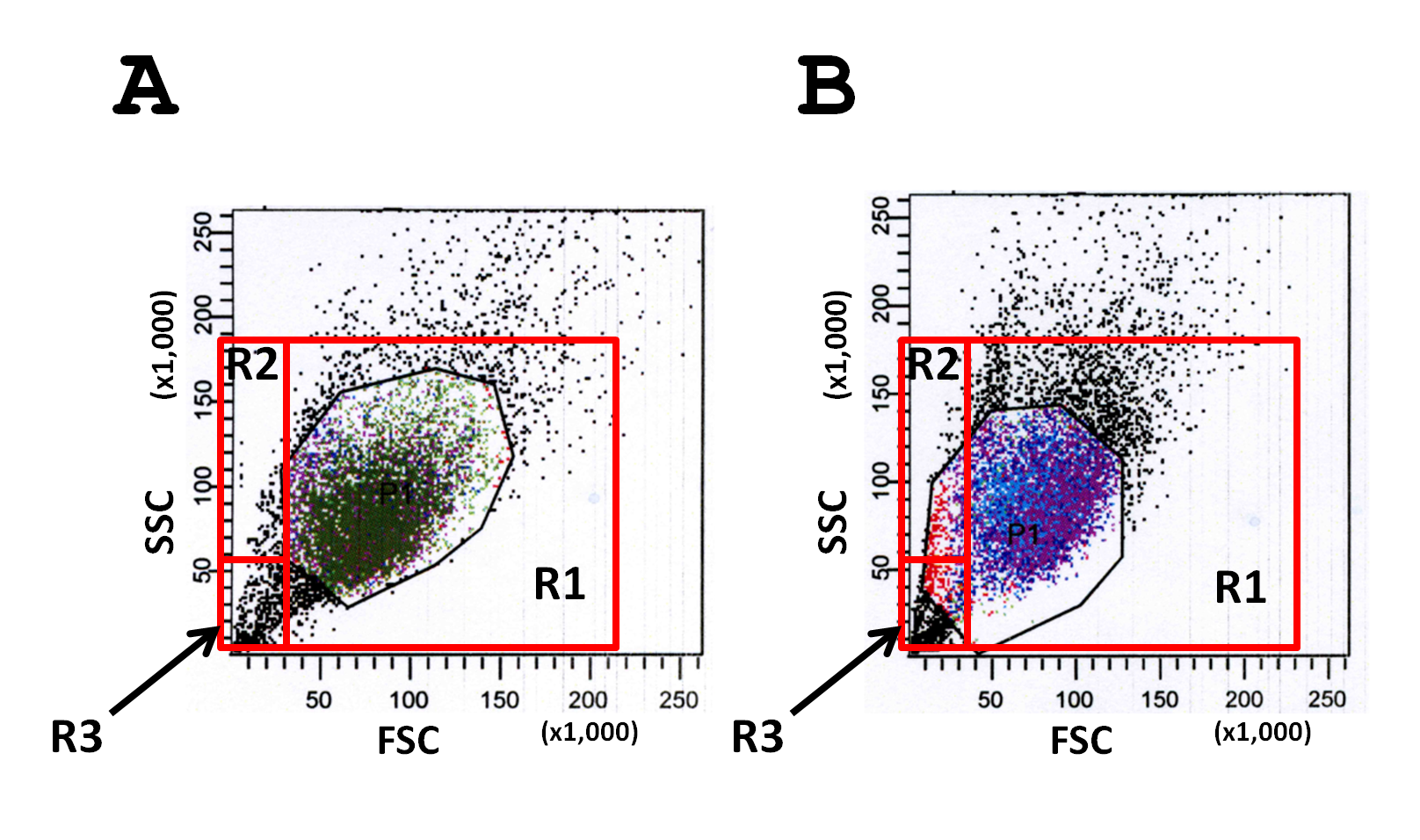

Supplement: Figure S1 — Selection gating for viable cells during FACS sorting of C1-EGFP (A) and D2SV-EGFP (B) transfected cells. Side scatter (SSC) and forward scatter (FSC) plots (A, B) were used to determine predicted viable cells for FACS sorting. Region 1 (R1) contains viable cells where as region 2 (R2) contains cells in early stages of apoptosis and region 3 (R3) contains dead cells. Cell shrinkage and nuclear condensations are two of the hallmarks of apoptosis. Cell shrinkage leads to a decrease in forward scatter (FSC) whereas nuclear condensation results in an increase in side scatter (SSC). EGFP-C1 and EGFP-cycD2SV sorted cells roughly contained same number of cells in regions 2 and 3. (TIF) [file pone.0053503.s001.tif]

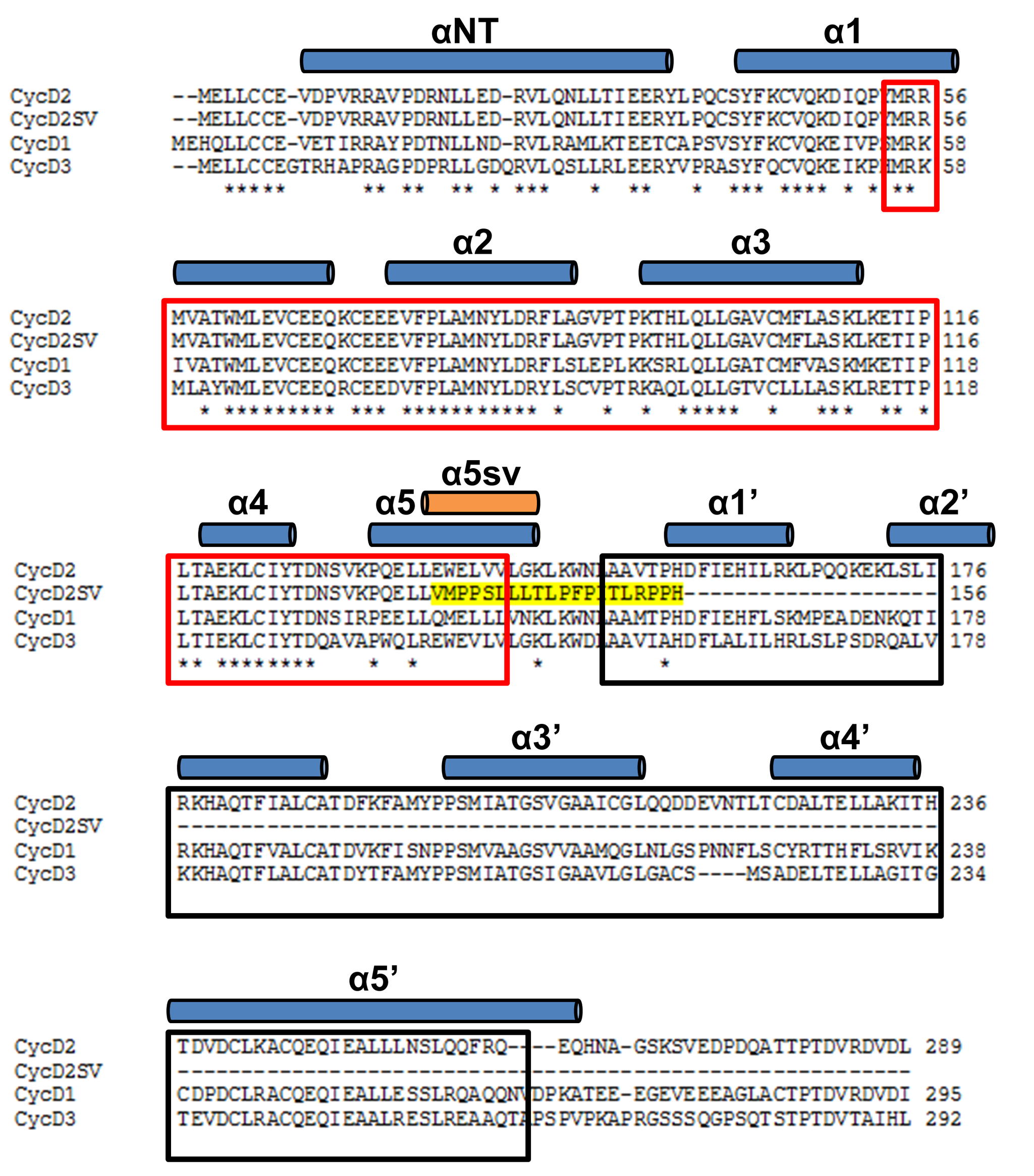

Supplement: Figure S2 — Sequence alignment of D-type cyclins and cycD2SV reveals important conserved domains. Cyclins in general contain two important cyclin folds, the N-terminal cyclin fold (red box) and the C-terminal cyclin fold (black box) each containing five alpha-helical domains. For clarity, the NT helical domains are labeled as α1-5 and the CT helical domains are labeled as α1′-5′. In general, cyclins also contain two additional NT and CT helical domains (αNT, αCT) located outside of the two cyclin folds. However, D-type cyclins appear to lack the αCT domain. The NT cyclin fold also known as the cyclin box is responsible for the association of cyclins with CDKs while the CT cyclin fold is thought to be responsible for binding of CDK activating kinase (CAK) and proper folding of the cyclin (GenBank: AAA37519.1). The cycD2SV CT sequence is highlighted in yellow. Helical domains are denoted by blue cylinders. The orange cylinder marks the helical domain (α5sv) present in the cycD2SV unique CT-domain. Asterix (*) denotes amino acids which are identical among all sequences. α denotes α-helix. (TIF) [file pone.0053503.s002.tif]

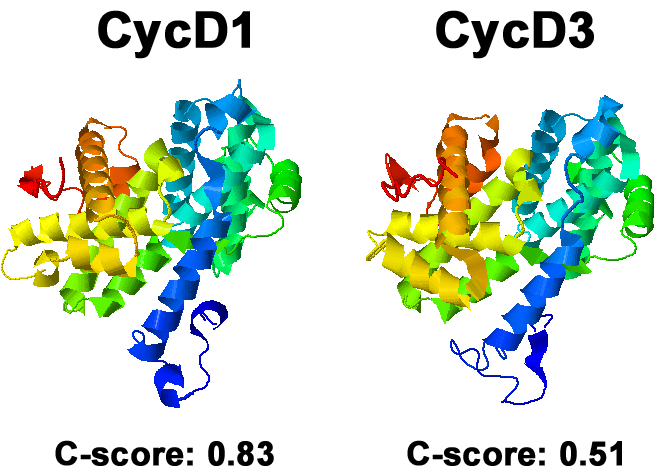

Supplement: Figure S3 — Three-dimensional (3D) protein structure predictions for cycD1 and cycD3. Protein structures were determined by the iterative threading assembly refinement (I-TASSER) server, an internet based 3D protein structure prediction engine. The N-terminus of the presented protein structures is denoted by blue and the C-terminus is denoted by red. (TIF) [file pone.0053503.s003.tif]
