# Supplementary material for: Characterization of Growth Suppressive Functions of a Splice Variant of Cyclin D2
Source: PLoS One. 2013 Jan 10;8(1):e53503. doi: 10.1371/journal.pone.0053503 (PMC3542336; doi:10.1371/journal.pone.0053503)
Supplement: Table S1 — Primers used for generation of DNA expression constructs. (DOC) [file pone.0053503.s004.doc]

| **Construct** | **Primer Name** | **Sequence 5’ – 3’** |
| --- | --- | --- |
| pEGFP-D2SV | *D2SVS4* | F GCCAGATCTAGTGGTGGCCGGCTGGCTATGGAGC |
|  | *D2altAS* | R CTTACAGTCTTGGTTAGTGTGGCGG |
| pcDNA- cycD2SV∆CTmyc | *D2ex1S* | F AGTGGTGGCCGGCTGGCTATGGAGC |
|  | *D2 1-136AS-Xba1* | R GCTCTAGACAGCAGCTCCTGGGGCTTCAC |
| pcDNA- cycD2SV  1-53myc | *SV 1-53 IF1* | F GTGGCGGCCGCTCGAGCGGCTGGCTATGGAGCTGCTG |
|  | *SV 1-53 IF2* | R GCCCTCTAGACTCGAGGTACGGTTGGATGTCCTTCTG |
| pcDNA- cycD2SV  54-136myc | cyc*D2 54-126S* | F CGGCTGGCTATGGAGATGCGCAGGATGGTG |
|  | *D2 1-136AS-Xba1* | R GCTCTAGACAGCAGCTCCTGGGGCTTCAC |

# Table S1. Primers used for generation of DNA expression constructs.

F= forward primer; R= reverse primer
