# Supplementary material for: Characterization of Growth Suppressive Functions of a Splice Variant of Cyclin D2
Source: PLoS One. 2013 Jan 10;8(1):e53503. doi: 10.1371/journal.pone.0053503 (PMC3542336; doi:10.1371/journal.pone.0053503)
Supplement: Table S2 — Cell cycle array analysis completed for C1-EGFP control and D2SV-EGFP sorted cells. (DOC) [file pone.0053503.s005.doc]

**Table S2. Cell cycle array analysis** completed for C1-EGFP control and D2SV-EGFP sorted cells.

|  |  | **2^-Avg.(∆CT)^** | |  |  |
| --- | --- | --- | --- | --- | --- |
| **Gene** | **Symbol** | **C1-EGFP** | **D2SV-EGFP** | **Mean of Fold Change** | ***p*-Value** |
| C-abl oncogene 1, receptor tyrosine kinase | ABL1 | 0.045507 | 0.046534 | 1.0226 | 0.896426 |
| Anaphase promoting complex subunit 2 | ANAPC2 | 0.011429 | 0.013394 | 1.1719 | 0.355643 |
| Anaphase promoting complex subunit 4 | ANAPC4 | 0.014301 | 0.014657 | 1.025 | 0.892721 |
| DIRAS family, GTP-binding RAS-like 3 | DIRAS3 | 0.000178 | 0.000187 | 1.0489 | 0.830631 |
| Ataxia telangiectasia mutated | ATM | 0.002082 | 0.001901 | 0.9131 | 0.76961 |
| Ataxia telangiectasia and Rad3 related | ATR | 0.014534 | 0.01276 | 0.878 | 0.459864 |
| BCL2-associated X protein | BAX | 0.436955 | 0.460448 | 1.0538 | 0.578846 |
| BRCA2 and CDKN1A interacting protein | BCCIP | 0.316927 | 0.296844 | 0.9366 | 0.735572 |
| B-cell CLL/lymphoma 2 | BCL2 | 0.004291 | 0.004532 | 1.0562 | 0.771185 |
| Baculoviral IAP repeat-containing 5 | BIRC5 | 0.011091 | 0.009363 | 0.8441 | 0.750298 |
| Breast cancer 1, early onset | BRCA1 | 0.030372 | 0.03056 | 1.0062 | 0.847403 |
| Breast cancer 2, early onset | BRCA2 | 0.003753 | 0.004585 | 1.2217 | 0.194956 |
| Cyclin B1 | CCNB1 | 0.875931 | 0.710929 | 0.8116 | 0.347278 |
| Cyclin B2 | CCNB2 | 0.191961 | 0.192257 | 1.0015 | 0.918471 |
| Cyclin C | CCNC | 0.120928 | 0.110934 | 0.9174 | 0.584678 |
| Cyclin D1 | CCND1 | 0.018143 | 0.011394 | 0.628 | 0.341384 |
| Cyclin D2 | CCND2 | 0.135424 | 0.13532 | 0.9992 | 0.952798 |
| Cyclin E1 | CCNE1 | 0.054242 | 0.060978 | 1.1242 | 0.264414 |
| Cyclin F | CCNF | 0.021726 | 0.022788 | 1.0489 | 0.728108 |
| Cyclin G1 | CCNG1 | 0.51963 | 0.512081 | 0.9855 | 0.884616 |
| Cyclin G2 | CCNG2 | 0.013405 | 0.012613 | 0.941 | 0.69836 |
| Cyclin H | CCNH | 0.078503 | 0.090943 | 1.1585 | 0.715719 |
| Cyclin T1 | CCNT1 | 0.05977 | 0.072349 | 1.2105 | 0.244277 |
| Cyclin T2 | CCNT2 | 0.017729 | 0.019656 | 1.1087 | 0.433431 |
| Cell division cycle 16 homolog (S. cerevisiae) | CDC16 | 0.125482 | 0.127725 | 1.0179 | 0.858039 |
| Cell division cycle 2, G1 to S and G2 to M | CDC2 | 0.512476 | 0.470123 | 0.9174 | 0.524907 |
| Cell division cycle 20 homolog (S. cerevisiae) | CDC20 | 0.50309 | 0.470123 | 0.9345 | 0.731918 |
| Cell division cycle 34 homolog (S. cerevisiae) | CDC34 | 0.015046 | 0.019884 | 1.3215 | 0.208111 |
| Cyclin-dependent kinase 2 | CDK2 | 0.140524 | 0.131316 | 0.9345 | 0.670209 |
| Cyclin-dependent kinase 4 | CDK4 | 0.409581 | 0.437628 | 1.0685 | 0.606063 |
| Cyclin-dependent kinase 5, regulatory subunit 1 (p35) | CDK5R1 | 0.003494 | 0.004075 | 1.1665 | 0.474335 |
| CDK5 regulatory subunit associated protein 1 | CDK5RAP1 | 0.002141 | 0.002646 | 1.2359 | 0.52246 |
| Cyclin-dependent kinase 6 | CDK6 | 0.030023 | 0.028712 | 0.9563 | 0.610107 |
| Cyclin-dependent kinase 7 | CDK7 | 0.05108 | 0.050805 | 0.9946 | 0.995433 |
| Cyclin-dependent kinase 8 | CDK8 | 0.028142 | 0.02767 | 0.9832 | 0.951884 |
| Cyclin-dependent kinase inhibitor 1A (p21Cip1) | CDKN1A | 0.182448 | 0.193148 | 1.0586 | 0.770827 |
| Cyclin-dependent kinase inhibitor 1B (p27Kip1) | CDKN1B | 0.027948 | 0.028316 | 1.0132 | 0.935082 |
| Cyclin-dependent kinase inhibitor 2A (p16) | CDKN2A | 0.127823 | 0.111191 | 0.8699 | 0.472093 |
| Cyclin-dependent kinase inhibitor 2B (p15) | CDKN2B | 0.024443 | 0.026298 | 1.0759 | 0.352026 |
| Cyclin-dependent kinase inhibitor 3 | CDKN3 | 0.154844 | 0.159812 | 1.0321 | 0.860987 |
| CHK1 checkpoint homolog (S. pombe) | CHEK1 | 0.088729 | 0.103745 | 1.1692 | 0.172583 |
| CHK2 checkpoint homolog (S. pombe) | CHEK2 | 0.057867 | 0.056112 | 0.9697 | 0.701998 |
| CDC28 protein kinase regulatory subunit 1B | CKS1B | 0.14717 | 0.136892 | 0.9302 | 0.739475 |
| CDC28 protein kinase regulatory subunit 2 | CKS2 | 0.584614 | 0.533827 | 0.9131 | 0.650498 |
| Cullin 1 | CUL1 | 0.068183 | 0.056112 | 0.823 | 0.615898 |
| Cullin 2 | CUL2 | 0.129608 | 0.124232 | 0.9585 | 0.815363 |
| Cullin 3 | CUL3 | 0.000934 | 0.000756 | 0.8098 | 0.327323 |
| DEAD/H (Asp-Glu-Ala-Asp/His) box polypeptide 11 (CHL1-like helicase homolog, S. cerevisiae) | DDX11 | 0.014908 | 0.013394 | 0.8985 | 0.944014 |
| Dynamin 2 | DNM2 | 0.006385 | 0.009363 | 1.4663 | 0.044266 |
| E2F transcription factor 4, p107/p130-binding | E2F4 | 0.030023 | 0.028645 | 0.9541 | 0.695106 |
| Growth arrest and DNA-damage-inducible, alpha | GADD45A | 0.00742 | 0.012184 | 1.6421 | 0.042191 |
| General transcription factor IIH, polypeptide 1, 62kDa | GTF2H1 | 0.127528 | 0.135633 | 1.0636 | 0.589076 |
| G-2 and S-phase expressed 1 | GTSE1 | 0.016089 | 0.013425 | 0.8344 | 0.597626 |
| Hect domain and RLD 5 | HERC5 | 0.024899 | 0.030419 | 1.2217 | 0.054532 |
| HUS1 checkpoint homolog (S. pombe) | HUS1 | 0.003149 | 0.003515 | 1.1164 | 0.79165 |
| Kinetochore associated 1 | KNTC1 | 0.044777 | 0.044433 | 0.9923 | 0.910348 |
| Karyopherin alpha 2 (RAG cohort 1, importin alpha 1) | KPNA2 | 0.247509 | 0.256041 | 1.0345 | 0.932005 |
| MAD2 mitotic arrest deficient-like 1 (yeast) | MAD2L1 | 0.541696 | 0.461513 | 0.852 | 0.50983 |
| MAD2 mitotic arrest deficient-like 2 (yeast) | MAD2L2 | 0.056939 | 0.055724 | 0.9787 | 0.856011 |
| Minichromosome maintenance complex component 2 | MCM2 | 0.178281 | 0.199498 | 1.119 | 0.418758 |
| Minichromosome maintenance complex component 3 | MCM3 | 0.458679 | 0.50737 | 1.1062 | 0.115046 |
| Minichromosome maintenance complex component 4 | MCM4 | 0.142815 | 0.155801 | 1.0909 | 0.500166 |
| Minichromosome maintenance complex component 5 | MCM5 | 0.084137 | 0.108401 | 1.2884 | 0.419207 |
| Antigen identified by monoclonal antibody Ki-67 | MKI67 | 0.260416 | 0.245044 | 0.941 | 0.752684 |
| Menage a trois homolog 1, cyclin H assembly factor (Xenopus laevis) | MNAT1 | 0.085115 | 0.0935 | 1.0985 | 0.493612 |
| MRE11 meiotic recombination 11 homolog A (S. cerevisiae) | MRE11A | 0.007317 | 0.007983 | 1.0909 | 0.925512 |
| Nibrin | NBN | 0.00166 | 0.001694 | 1.0202 | 0.696459 |
| Proliferating cell nuclear antigen | PCNA | 0.728107 | 0.772592 | 1.0611 | 0.584027 |
| RAD1 homolog (S. pombe) | RAD1 | 0.03339 | 0.030701 | 0.9195 | 0.630274 |
| RAD17 homolog (S. pombe) | RAD17 | 0.041298 | 0.035512 | 0.8599 | 0.350128 |
| RAD51 homolog (RecA homolog, E. coli) (S. cerevisiae) | RAD51 | 0.001429 | 0.001363 | 0.9541 | 0.849772 |
| RAD9 homolog A (S. pombe) | RAD9A | 0.026502 | 0.028121 | 1.0611 | 0.699695 |
| Retinoblastoma 1 | RB1 | 0.010813 | 0.014489 | 1.34 | 0.645515 |
| Retinoblastoma binding protein 8 | RBBP8 | 0.052759 | 0.046534 | 0.882 | 0.458713 |
| Retinoblastoma-like 1 (p107) | RBL1 | 0.052153 | 0.072349 | 1.3872 | 0.095527 |
| Retinoblastoma-like 2 (p130) | RBL2 | 0.011066 | 0.011083 | 1.0015 | 0.842176 |
| Replication protein A3, 14kDa | RPA3 | 0.262834 | 0.247891 | 0.9431 | 0.747903 |
| SERTA domain containing 1 | SERTAD1 | 0.014908 | 0.01993 | 1.3369 | 0.420479 |
| S-phase kinase-associated protein 2 (p45) | SKP2 | 0.335772 | 0.330131 | 0.9832 | 0.927152 |
| SMT3 suppressor of mif two 3 homolog 1 (S. cerevisiae) | SUMO1 | 0.096203 | 0.095025 | 0.9878 | 0.882802 |
| Transcription factor Dp-1 | TFDP1 | 0.041394 | 0.037364 | 0.9026 | 0.858238 |
| Transcription factor Dp-2 (E2F dimerization partner 2) | TFDP2 | 0.049798 | 0.05284 | 1.0611 | 0.816079 |
| Tumor protein p53 | TP53 | 0.108484 | 0.116719 | 1.0759 | 0.817862 |
| Ubiquitin-like modifier activating enzyme 1 | UBA1 | 0.121769 | 0.07577 | 0.6222 | 0.284865 |
